# Characterization and comparative profiling of the small RNA transcriptomes in two phases of locust

**DOI:** 10.1186/gb-2009-10-1-r6

**Published:** 2009-01-16

**Authors:** Yuanyuan Wei, Shuang Chen, Pengcheng Yang, Zongyuan Ma, Le Kang

**Affiliations:** 1 State Key Laboratory of Integrated Management of Pest Insects and Rodents, Institute of Zoology, Chinese Academy of Sciences, Datun Road, Chaoyang District, Beijing 100101, PR China

## Abstract

**Background:**

All the reports on insect small RNAs come from holometabolous insects whose genome sequence data are available. Therefore, study of hemimetabolous insect small RNAs could provide more insights into evolution and function of small RNAs in insects. The locust is an important, economically harmful hemimetabolous insect. Its phase changes, as a phenotypic plasticity, result from differential gene expression potentially regulated at both the post-transcriptional level, mediated by small RNAs, and the transcriptional level.

**Results:**

Here, using high-throughput sequencing, we characterize the small RNA transcriptome in the locust. We identified 50 conserved microRNA families by similarity searching against miRBase, and a maximum of 185 potential locust-specific microRNA family candidates were identified using our newly developed method independent of locust genome sequence. We also demonstrate conservation of microRNA*, and evolutionary analysis of locust microRNAs indicates that the generation of miRNAs in locusts is concentrated along three phylogenetic tree branches: bilaterians, coelomates, and insects. Our study identified thousands of endogenous small interfering RNAs, some of which were of transposon origin, and also detected many Piwi-interacting RNA-like small RNAs. Comparison of small RNA expression patterns of the two phases showed that longer small RNAs were expressed more abundantly in the solitary phase and that each category of small RNAs exhibited different expression profiles between the two phases.

**Conclusions:**

The abundance of small RNAs in the locust might indicate a long evolutionary history of post-transcriptional gene expression regulation, and differential expression of small RNAs between the two phases might further disclose the molecular mechanism of phase changes.

## Abstract 2

High-throughput sequencing of the small RNA transcriptome of locust reveals differences in post-transcriptional regulation between solitary and swarming phases and provides insights into the evolution of insect small RNAs.

## Background

Regulation of gene expression can occur at both transcriptional and post-transcriptional levels. In recent years, the discovery of numerous small RNAs has increased interest in post-transcriptional gene expression regulation during development and other biological processes. Small RNAs include several kinds of short non-coding RNAs, such as microRNA (miRNA), small interfering RNA (siRNA), and Piwi-associated RNA (piRNA), which all regulate gene expression at the post-transcriptional level. Typically, miRNAs are approximately 22 nucleotide small-RNA sequences [1] that play key roles in many diverse biological processes, including development, viral defense, metabolism, and apoptosis [2-5]. The 'seed' region, located at miRNA nucleotides 2-8 [6], is the most important sequence for interaction with mRNA targets. There are two other important non-coding RNAs: endogenous siRNA (endo-siRNA) and piRNA. Endo-siRNA is derived from double-stranded RNA to guide RNA interference. Much of the research on endo-siRNAs has been done in plants [7], but recently endo-siRNAs derived from transposons and mRNAs in flies have also been identified [8]. These findings indicate that endo-siRNAs may play a broader role in all organisms. A new class of small RNAs, piRNA, was discovered two years ago. piRNAs, 23-30 nucleotides in length, interact with PIWI proteins and repress the expression of selfish genetic elements, such as transposons, in the germ line [9,10].

Insects comprise the largest group of metazoans, and previous studies have shown that small RNAs are involved in a significant number of biological processes in them [11]. Many small RNAs have been identified in insects whose whole genome sequences are available, including the fruit fly, bee, mosquito, and silkworm. These insects are all holometabolous, meaning that they go through the complete four stages of metamorphism. Another important group of insects are hemimetabolous insects, which undergo an incomplete metamorphism, bypassing the pupa stage. In this group of insects, no research on small RNAs has been carried out. Studies on small RNAs in very different groups of insects are important for understanding the evolution of post-transcriptional gene expression regulation, and gaining specific information from the hemimetabolous group represents a unique opportunity to examine species with an analogous, but modified, developmental process. Combined with the holometabolous group, the study of small RNAs in the hemimetabolous group, including several ancient orders of insects, could aid in understanding the whole picture of evolution and function of small RNAs in insects.

The migratory locust (*Locusta migratoria*) is a typical hemimetabolous insect within the family Acrididae and is a worldwide, highly prevalent agricultural pest causing hundreds of millions of dollars worth of damage every year. The locust has also been used in research as a model organism for the study of developmental, physiological, immune, and neural pathways, as well as others [12]. Additionally, as compared to the fruit fly, the locust is a far more primitive insect, making it an excellent model for studying evolution.

A great deal of work has been carried out specifically on the ability of the locust to change phases from solitary to gregarious (in the latter phase, locusts form swarms that cause devastation of crops). Phase transition, as a phenotypic plasticity in response to population density changes, is one of the most interesting behavioral phenomena of the locust, and is linked with changes in morphology, behavior, reproduction, endocrine balance, and disease resistance, all of which include many changes at the molecular level that are potentially involved in both transcriptional [13] and post-transcriptional regulation of gene expression. Given that small RNAs are known to be a key component in post-transcriptional gene expression regulation in a variety of organisms, information on the presence and activities of small RNAs in the locust would be particularly useful. The locust, however, currently lacks any substantial genome sequence data. Thus, the available expressed sequence tags (ESTs) [13,14] provide the only basis for small RNA annotation. It is possible to identify the precursors of miRNAs and endogenous siRNAs via alignment to ESTs [15,16]. The identification and comparison of small RNAs in the gregarious and solitary phases can aid in understanding the mechanisms underlying their different biological processes, especially phase transition. Furthermore, differences in small RNAs between the two phases might provide clues about how to control locust plagues throughout the world by designing artificial siRNAs, thus saving a huge number of crops every year.

For this study, because there is no whole genomic information available, we utilized the new high-throughput sequencing method (Illumina Genome Analyzer), instead of computational approaches, to characterize locust small RNAs, and developed a new method to predict locust-specific miRNAs. We further compared the small RNA characteristics and expression patterns between the gregarious and solitary phases.

## Results

### High-throughput sequencing of small RNAs

**Figure 1:**
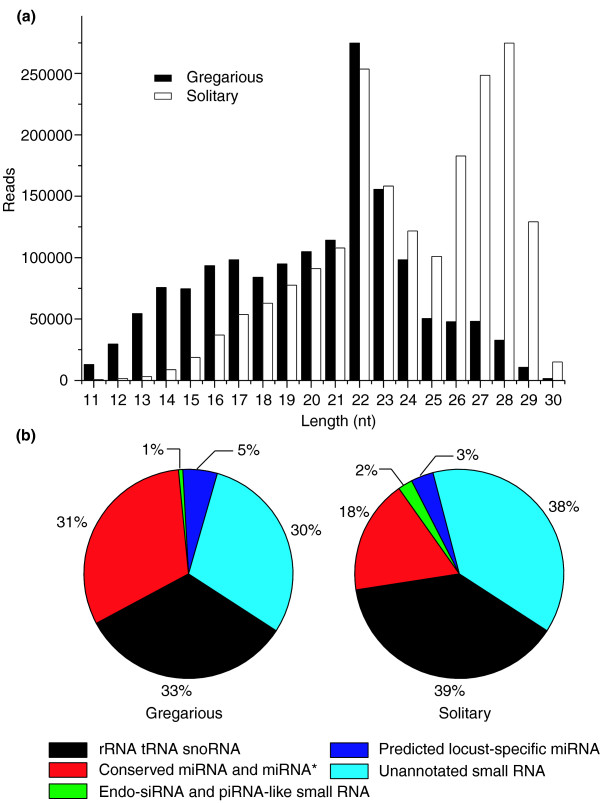
Length distribution and composition of the small RNA libraries in gregarious and solitary locusts. Nt, nucleotides.

To survey small RNAs in the locust, we used Illumina sequencing technology on libraries of small RNAs from the gregarious and solitary phases [GEO:GSE12640]. We obtained 1,566,242 reads from the gregarious library and 1,949,248 reads from the solitary library after discarding the empty adapters. Generally, length distribution of small RNAs in two phase libraries is different (Figure 1a; also see the section 'Different expression profiles of small RNAs in the two phases' below). After discarding low-quality sequences, sequences shorter than 18 nucleotides, and single-read sequences, 895,554 and 1,377,859 reads, for the gregarious and solitary phases, respectively, remained for analysis. After comparing the small RNA sequences with the locust EST database [13,14] as well as the *Drosophila melanogaster *rRNA, tRNA and snoRNA database [17], sequences that came from these types of RNAs (Figure 1b) were removed. The remaining sequences were clustered based on sequence similarity because related sequences probably came from the same precursor as cleavage by RNase III enzymes was imprecise. We determined that the sequence with the dominant number of reads in a cluster was likely to be the real sequence due to its relatively high expression level, and these sequence clusters were further analyzed.

### Conserved microRNAs

We identified 55 miRNA sequences, belonging to 50 families (Table S1 in Additional data file 3), in the migratory locust by BLAST against the miRBase v11.0 [18]. Most of the 50 miRNA families share the same 'seed' regions (the 5' region important for target recognition) [6] in the locust and other insects. However, locust miR-10 and miR-79 (lmi-miR-10 and lmi-miR-79) have very different 5' ends, thus changing their 'seed' region, compared with miR-10 and miR-79 of the other four insect species studied. For locust miR-79, the mature sequence has an additional adenosine at the 5' end (Figure S1 in Additional data file 3), similar to that of the *Caenorhabditis elegans *miR-79 (cel-miR-79). Although in most cases the key 'seed' site of the miRNA is nucleotides 2-8 [6,19], the 8-mer seed site of *D. melanogaster *miR-79 (dme-miR-79) has been validated as being at nucleotides 1-8 [6], which is the same as locust miR-79 nucleotides 2-9. This indicates that the additional adenosine at the 5' end of lmi-miR-79 possibly does not lead to different targets in the locust and fly.

For lmi-miR-10, much like lmi-miR-79, the mature sequence in the locust has an additional nucleotide at the 5' end, in this case a uridine (Figure S1 in Additional data file 3), which is the same as the miR-10 of non-insect organisms. Previous studies have demonstrated that miR-10 in both species that do and do not have an extra U have similar targets [20]. Although lmi-miR-79 and lmi-miR-10 of the locust have an extra nucleotide at the 5' end compared to those of the fruit fly, they still have the same 'seed' sequences, which may potentially regulate similar targets.

### Conservation of miRNA*

Although mature miRNA and miRNA* (the miRNA:miRNA* duplex) are complementary, their base-pairing is imperfect in the presence of compensatory substitutions (for example, C-G to U-G), and the miRNA* is generally less stable than the mature miRNA [21]. Analysis of miRNA and miRNA* species in the miRNA database [18] indicated that miRNA* is less conserved than miRNA (data not shown). However, we found the homologs of several *D. melanogaster *miRNA* (miR-iab-4, miR-8, miR-9a, miR-10, miR-210, miR-276, miR-281, and miR-307; Table S2 in Additional data file 3) in the locust library, indicating conservation of these miRNAs* between the locust and the fruit fly.

**Figure 2:**
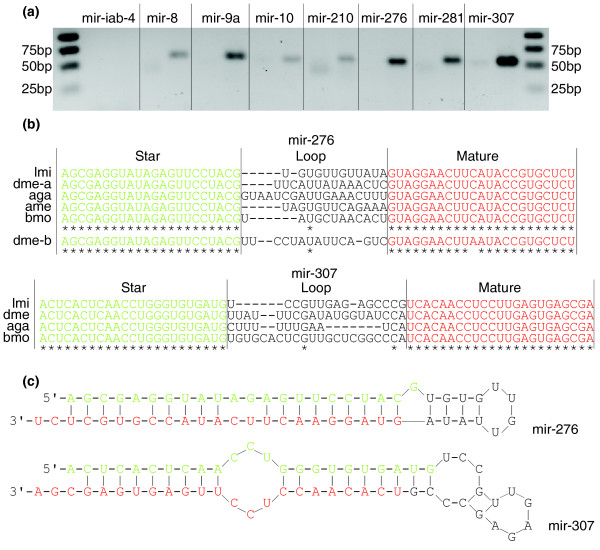
Conservation of miRNA* in the locust. **(a) **Electrophoretic analysis of PCR products amplified by the primer pairs designed on the basis of predicted miRNA* as based on a similarity to fruit fly miRNA* and their corresponding mature miRNAs. For each miRNA, the left lane is the negative control and the right lane is the positive result. **(b) **Two examples of precursor sequences of seven conserved miRNAs that have a conserved star sequence. The alignment of mir-276 and mir-307 in different insects shows high conservation of their miRNA*. The green nucleotides represent miRNA star sequence and the red represent mature miRNA sequence. The asterisks indicate the conserved sites among these species. **(c) **Hairpin structures of the mir-276 and mir-307 precursors of the locust. aga, *A*. *gambiae*; ame, *A*. *mellifera*; bmo, *B*. *mori*; dme, *D*. *melanogaster*; lmi, *L*. *migratoria*.

To test whether the locust miRNA* and their corresponding mature miRNA sequences came from the same precursors, we used a PCR-based method to confirm the relationship between the miRNA and its miRNA*. If the miRNA and its miRNA* came from the same precursor, we should be able to amplify 55-70 bp fragments from the genomic DNA. As expected, we amplified 55-70 bp products from all the miRNAs with the exception of mir-iab-4 (Figure 2a), and the sequences of the PCR products confirmed the matches between miRNAs* and mature miRNAs (Table S2 in Additional data file 3). We could not amplify the expected products of mir-iab-4, although we repeatedly performed the PCR experiments; the two sequences probably do not comprise the canonical miRNA precursor in the locust.

We used the sequences of the amplified products of the conserved miRNA precursors to predict their secondary structure using mfold [22,23], and all seven sequences could be properly folded into the typical hairpin structure (Figure 2c), again indicating that the miRNA pairs came from the same precursor and could properly fold into the pre-miRNA-like hairpin for further processing. Taken together, these data indicate that, in addition to conservation of mature miRNAs, some of the locust miRNA* are also highly conserved in different lineages (Figure 2b). That the miRNA* are conserved across several lineages indicates a possible role of miRNA* in regulating gene expression, which was previously reported in flies [24].

Since the locust and fruit fly separated about 350 million years ago [25], it is striking that the 22-nucleotide miRNA* has little sequence divergence between the two species. Moreover, in the case of lmi-mir-10, a greater number of reads (two-fold more abundant) was generated by the star form. For lmi-mir-8 and lmi-mir-276, thousands of their star reads were presented in the library (Figure S2 in Additional data file 3). These findings also implicated a functional role of miRNA* in regulating gene expression.

### Identification of locust-specific miRNA families

In an attempt to discover locust-specific miRNA families, we integrated the data from the locust small RNA libraries we created with those of the locust EST database [13,14]. This, however, did not provide any significant findings (see Materials and methods), likely because of the low coverage of the locust EST database. Given that no methods were available to identify locust lineage-specific miRNA families in the absence of locust genomic information [26,27], we developed a new method that is based on high-throughput sequencing but does not require the presence of whole genome sequence data (see Materials and methods).

**Figure 3:**
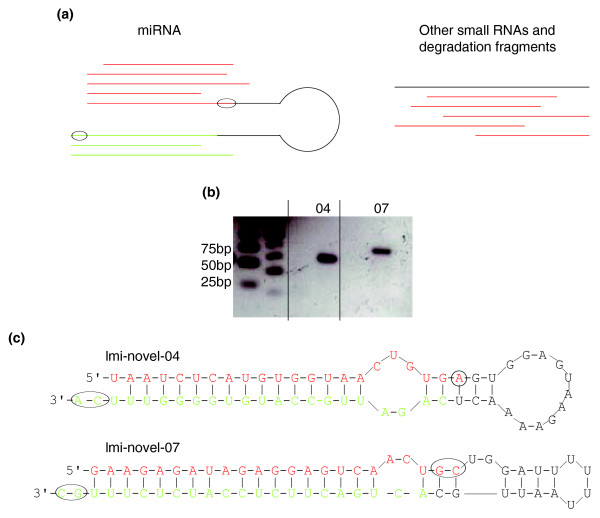
Principles of locust-specific miRNA prediction and examples of the secondary structure of locust-specific miRNA precursors. **(a)** The features of miRNA and other small RNAs. Left side: the red and green lines represent the mature miRNA and miRNA*, respectively, which can be found in the same small RNA library sequenced by high-throughput sequencing in most cases. The black circles show the 1-2 nucleotide 3' overhang of miRNA:miRNA* duplex. Right side: inconsistency at the 5' ends of other small RNAs and the degradation fragments. **(b) **Electrophoretic analysis of PCR products of lmi-novel-04 and lmi-novel-07, showing the expected length of 55-70 nucleotides. **(c) **The secondary structures of lmi-novel-04 and lmi-novel-07. The red sequence represents mature miRNA and the green represents miRNA*. The black circles indicate 1-2 nucleotide 3' overhangs.

We obtained 185 miRNA duplex-like pairs (Figure 3a; Table S3 in Additional data file 3 shows the sequences with the dominant reads, potential miRNA candidates, in the pairs). If these pairs were true miRNA duplexes, 55-70 bp fragments should be amplified from the locust genomic DNA using primers designed according to the duplexes. To test the validity of our method to identify species-specific miRNAs, we amplified corresponding fragments from locust genomic DNA for 24 of our predicted candidate duplexes. Using this method we obtained amplified fragments of expected length from 13 out of the 24 candidates (Figure 3b and Table 1), indicating that about half of the predicted candidates may be canonical miRNA duplexes of which the strand with more reads should be mature miRNA and the other strand should be miRNA*.

**Table 1:** Validated locust-specific miRNAs

miRNA family	Mature miRNA sequence (5'-3')	Length*	miRNA star sequence (5'-3')
lmi-novel-01	UCAGGAAAUCAAUCGUGUAAGU	22	UUACACAGCUGGUUUCCUGGGA
lmi-novel-02	UGAAGCUCCUCAUAUCUGACCU	22	GUGAGAUGUGAUGAGCUUCACU
lmi-novel-03	UAAGCUCGUCUUUCUGAGCAGU	22	UCUUCGGAGGCGUGGGUAUCCC
lmi-novel-04	UAAUCUCAUGUGGUAACUGUGA	22	CAGAUUGCCAUGUGGGGUUUCA
lmi-novel-05	AGCAUGAUCAGUGGCAUGAAUU	22	UUCGUGUGACUGCUCAUGCAAC
lmi-novel-06	AUGGUGUCAGGAAUAUGAGUCG	22	ACACAUAUUCCUGAUACUGACA
lmi-novel-07	GAAGAGAUAGAGGAGUCAACUGC	23	ACUGACUUCUCCAUCUCUUUGC
lmi-novel-08	CUGAAGUCACACGAGAGCGCCGU	23	CGCUCUCGUGUGACGUCAGGCA
lmi-novel-09	UUAUUCUGUCCGUGCCUCGAAA	22	UUUGGCAGGUGGGCAGAAUAUGU
lmi-novel-10	GUAGGCCGGCGGAAACUACUUG	22	AGGGGUUUCUUUCGGCCUCCAG
lmi-novel-11	AUGAGCAAUGUUAUUCAAAUGG	22	AUUUGAAUAUCAUUGCACAUUG
lmi-novel-12	UGAUGCUGCAGGAGUUGUUGUGU	23	AUGGUAACCCUUGAGGAGUCUUG
lmi-novel-13	ACUGACUGCCCUAUUUCUUUGC	22	GAAGAGAUAGGACAGUCAAUCU

*Length of mature miRNAs in nucleotides.

We sequenced 8 of the 13 amplified products and, using mfold [22,23], were able to confirm the ability of the 8 products to accurately fold in the typical hairpin structure of miRNA precursors (Figure 3c). For the 185 novel miRNA family candidates we predicted, we could not identify homologs in the *Drosophila *genome, indicating that they are probably species-specific families.

### miRNA expression patterns

High-throughput sequencing is not only a good tool for identifying small RNAs, it can also provide information about their expression levels. Compared with other small RNAs, miRNAs make up a larger proportion of the locust small RNA libraries (Figure 1b), indicating that miRNAs are the main kind of small RNAs involved in gene expression regulation in the locust. However, our libraries are made up of a mixture of different tissue samples at different developmental stages, so it is possible that the proportion of miRNAs to other small RNAs could vary in different tissues or developmental stages.

Some of the miRNAs we identified had more than one thousand reads, while others had fewer than ten (Figure S2 in Additional data file 3). Reads of the most abundant miRNAs are about 10,000-fold higher than those of the scarce miRNAs. Such extreme variation can provide some basic insight into the function of these miRNAs. The most abundant miRNA is mir-1, which had approximately 163,143 reads in the gregarious library and 135,794 in the solitary library. As a muscle-specific miRNA [28], mir-1 is the most abundant given its broad range of expression in different developmental stages and the high proportion of muscle tissues in the locust. As with mir-1, the miRNAs that have more reads should be expressed during most developmental stages, while those having fewer reads, such as mir-210 and lmi-novel-01 (Figure S2 in Additional data file 3), should be expressed in a much narrower range. It is likely that the expression of those exiguous miRNAs is developmentally related.

As miRNA abundance is linked to the extent of conservation [16,20], conserved miRNAs in the locust comprise more than 80% of the total miRNA reads we examined. The locust-specific miRNAs were expressed at a significantly lower level than those in conserved families (Wilcoxon rank-sum test, *p *< 1.0 × 10^-6^).

### Target prediction of miRNAs

**Figure 4:**
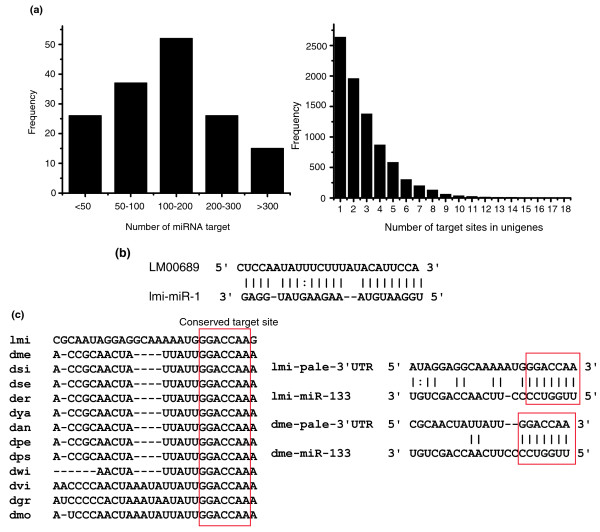
Target prediction of locust miRNAs. **(a) **Left side: distribution of target number of locust miRNAs. Right side: distribution of target site number of the unigenes. **(b) **Presumable pairing between lmi-miR-1 and LM00689 with highest score predicted by miRanda. **(c) **Conservation of mir-133 target site in the *pale *gene of locust (lmi) and 12 *Drosophila *species, and presumable pairing between miR-133 and the *pale *gene. The red boxes indicate conserved target sites of miR-133 in 3' UTR sequences of *pale*.

In animals, although miRNAs have been shown to repress the expression of their targets by binding to sequences in the 3' untranslated region (UTR) in most cases [29,30], both computational and experimental evidence show the existence of miRNA-binding sites in protein coding regions [31-34]. To identify potential targets of locust miRNAs, we searched unigene sequences from locust ESTs using miRanda 3.1 [35] because there is no 3' UTR database available (see Materials and methods). We found 8,212 unigenes targeted by 157 miRNAs (50 conserved miRNAs plus 7 conserved miRNA* plus the most abundant 100 locust-specific miRNA candidates predicted). All miRNAs have more than one predicted target, and some of the miRNAs even have more than 200 (Figure 4a). Similarly, some unigenes have more than one miRNA target site (Figure 4a). On average, every miRNA targets 147.5 unigenes and, conversely, every unigene is targeted by 2.8 miRNAs. We think that the higher the score given by miRanda, the more reliable the predicted results. The highest score for predicted targets was for LM00689, which is a potential target of lmi-miR-1 (Figure 4b). LM00689 is similar to the *ciboulot *gene of fruit fly, which encodes an actin binding protein and plays a major role in axonal growth during *Drosophila *brain metamorphosis [36].

We also found that some unigenes that had significant differences at the expression level between the gregarious and solitary phases were targeted by miRNAs. Although these genes may be regulated at the transcriptional level, it is possible that miRNAs play roles in regulating their expression. For example, microarray results in our lab show that the locust homolog of the *Drosophila *gene *pale *has significant differences in its expression levels between the two phases (Z Ma *et al*., unpublished). We found that the 3' UTR sequence of locust *pale *contains a target site of lmi-miR-133 (we got the 3' UTR sequences of *pale *in locust by 3' rapid amplification of cDNA ends (RACE); see Materials and methods; Figure 4c). We also found that in addition to the locust, 12 *Drosophila *species also have conserved target sites of miR-133 in the 3' UTR sequences of the *pale *gene [17,20,32] (Figure 4c), indicating the strong possibility of miR-133 regulating the expression of *pale *at the post-transcriptional level. Therefore, miR-133 may contribute to the different expression of *pale *between the gregarious and solitary phases (see Discussion).

### The phylogenetic evolution of miRNAs

**Figure 5:**
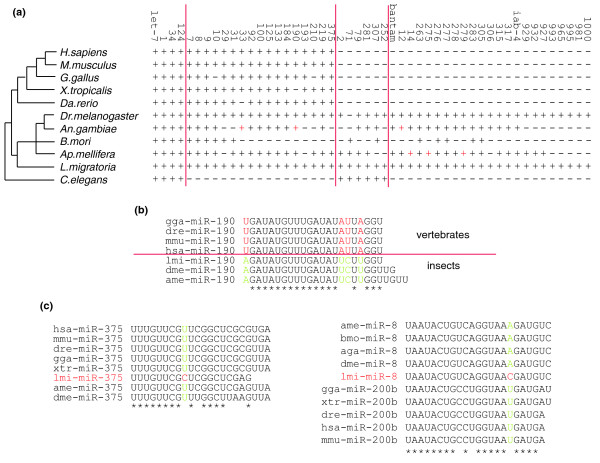
Phylogenetic evolution of locust conserved miRNA families. **(a)** Phylogenetic distribution of 50 conserved miRNA families of the locust. A plus (+) symbol indicates this miRNA family is found in the species named on the left, and a minus (-) symbol means it is absent in that species. A red plus symbol means this miRNA family can not be found in any database, but was found by our search in the corresponding species genome. **(b) **An example of clade-specific conserved miRNAs based on sequence substitutions. The red nucleotides indicate the positions that are the same among vertebrates but different from insects, which are shown in green. Vertebrates and insects can be easily separated according to sequence differences in their miR-190, showing the different sequence features of conserved miRNAs in different clades. The asterisks indicate the conserved sites among these species. **(c) **Two conserved miRNA families whose sequences are unique in the locust (lmi). The red nucleotide shows the locust-specific position that is different from any other species. The asterisks indicate the conserved sites among these species.

We sorted the 50 conserved families identified in the locust into 4 groups based on their phylogenetic distribution (Figure 5a). Four families (let-7, mir-1, mir-34, and mir-124) are present in insects, vertebrates, and nematodes; 17 families are present in insects and vertebrates, but not nematodes; 6 families are restricted to invertebrates (insects and nematodes); and the remaining 23 families are insect-specific.

Categorization of conserved miRNAs indicates that the innovation of miRNAs in the locust is concentrated along three branches of the phylogenetic tree leading to bilaterians, coelomates, and insects. Different conserved miRNAs in the locust have different ages. Some of them are from ancient families (for example, mir-1) and some appear to be much younger (for example, insect-specific miRNA families). Such age differences indicate that there is an ongoing process of miRNA evolution and it is possible that the insect lineage gave birth to the insect-specific miRNAs. Previous work in *Drosophila *has also indicated that the birth and death of miRNA families is a common phenomenon in insect evolution [37].

Although the 50 miRNA families in the locust are highly conserved throughout widely divergent animal taxa, there are lineage-specific sequence substitutions in most of these families that are present in both vertebrates and insects. Based on their characteristic sequences in different lineages, we divided these families into five categories (Table 2); in doing this we disregarded the deletion of nucleotides at the end of the miRNAs due to the inability to always accurately predict the termini of mature miRNAs. If a miRNA family had more than one of its members in certain species, we chose the member most similar to those in other species for use in categorizing because it may be an ancient member of the family. Families in category I have identical sequences in all observed species. Category II includes those families with small differences between invertebrates and vertebrates. Category III is made up of miRNA families that have identical sequences in all but one of the observed species. Category IV contains miRNAs with multiple variances in different lineages. Category V contains only one miRNA family (mir-1), which is identical in worms and vertebrates but not in insects.

**Table 2:** Categories of conserved miRNA families common in vertebrates and insects according to their sequences

Category	miRNA families
I	mir-7, mir-9, mir-124, mir-133, mir-219
II	mir-92, mir-190
III	let-7, mir-10, mir-33, mir-100, mir-184
IV	mir-8, mir-29, mir-31, mir-34, mir-125, mir-193, mir-210, mir-375
V	mir-1,

Despite the short sequences of mature miRNAs, the major clades are well separated due to substitutions in categories II to IV (Figure 5b), indicating that these miRNAs may have clade-specific functions. Scanning miRNA families in these categories, we identified two families, mir-8 and mir-375, by which the locust can be separated from other species (Figure 5c). Substitutions in mature miRNAs may lead to changes of targets, so it is likely that locust mir-8 and mir-375 have different modes of gene regulation in the locust.

### Endogenous siRNAs

We found that 26,519 reads matched the sense strand of ESTs and 11,596 reads matched the antisense strand [13,14] in the gregarious and solitary phase libraries. We classified the small RNAs matching the antisense strand as candidate endo-siRNAs (see Materials and methods; Additional data file 1).

**Figure 6:**
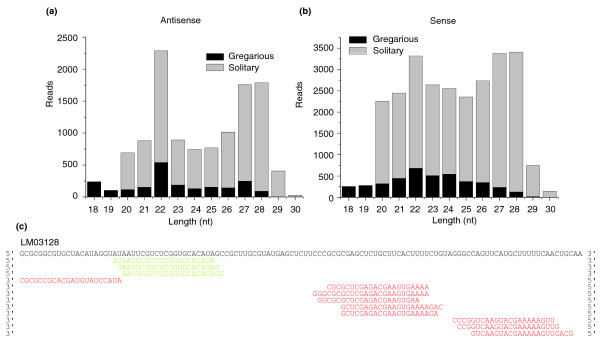
Small RNAs that match to EST sequences perfectly. **(a) **The length distribution of the reads matching antisense strands of ESTs. **(b) **The length distribution of the reads matching sense strands of ESTs. **(c) **Portions of one locust EST aligned with small RNA reads that matched the sense (green) and antisense (red) strands.

The proportion of endo-siRNAs in the small RNA libraries of locust is much lower than that of miRNAs (Figure 1b). However, because of incomplete mRNA sequence information in the locust EST database, the actual number of endo-siRNAs is likely to be higher. To gain greater understanding of the features of locust endo-siRNAs, we carried out additional analysis of these RNAs. Endo-siRNA length showed a major peak at 22 nucleotides, the same as miRNAs (Figure 6a); however, these small RNAs did not have a tendency to begin with uracil, a common feature of miRNA (data not shown). This provided additional evidence that these 22-nucleotide small RNAs were endo-siRNAs rather than miRNAs. In addition to the major peak at 22 nucleotides, there was also a minor peak at 27-28 nucleotides in endo-siRNAs. For small RNAs coming from sense strands of ESTs, in addition to a main peak at 22 nucleotides, there were also peaks at 27 nucleotides and 28 nucleotides (Figure 6b). An example of ESTs, aligned with small RNA reads that match the sense and antisense strands, is shown (Figure 6c). Also shown in the endo-siRNA sequences is the high variation at the 5' end of endo-siRNA clusters and their differences from the miRNA 5' ends (Figures 3a and 6c), again indicating their having a different role in gene silencing from miRNAs.

### Small RNAs derived from transposons

About 20% (8,353 reads) of the small RNAs with a perfect match with ESTs were derived from transposons. Previous research has shown that transposons can generate two kinds of small RNAs: endo-siRNA and piRNA [8], which are 22-23 and 23-29 nucleotides long, respectively. Therefore, the shorter sequences derived from transposons may be endo-siRNAs and the longer may be piRNAs (Additional data file 2).

There are a variety of transposons that could generate small RNAs regardless of whether they are siRNAs or piRNAs (Figure S3 in Additional data file 3), which may indicate the presence of a broad range of small RNAs for silencing these selfish genetic elements. Analysis of the transposons we observed indicated that long interspersed elements (LINEs) were the dominant class producing small RNAs (approximately 60% of the transposon-derived small RNAs). CR1 and RTE-BovB are the dominant subtypes generating small RNAs (approximately 34% of the transposon-derived small RNAs). As more transposon sequence information in the locust becomes available, we expect there will be additional transposon-derived small RNAs identified, which will give greater understanding of the impact of these elements on genome evolution of the locust and related species.

### Classification of the rest of the small RNAs

The rest of the sequences in the locust small RNA libraries remained unannotated. Most of the unannotated sequences in the gregarious library were 22 and 23 nucleotides long and commonly began with uracil (Figure S4a in Additional data file 3). We expected that these were miRNAs missed in our search process, and thus suspected that these 22- and 23-mer RNAs included additional locust-specific miRNAs. In order to identify the potential miRNA candidates in the remaining 22- and 23-nucleotide sequences, we analyzed their 5' ends to determine whether they were similar in features to the miRNA 5' terminus (Figure 3a; see Materials and methods). Our data showed that 10,161 reads (1,025 clusters, 1,275 unique sequences) had a standard miRNA-like 5' end and, therefore, probably were miRNAs (Table S4 in Additional data file 3).

There were also longer small RNAs (26-29 nucleotides) that generally began with uracil, especially in the solitary library (Figure S4b in Additional data file 3). Their features indicated that these 26-29-mer small RNAs might be of the piRNA class of small RNAs. Therefore, we analyzed the sequences of these small RNAs to look for the presence of an adenine at position 10, a common feature of piRNAs [9] (Figure S4c in Additional data file 3). Interestingly, although the 26-27-mer small RNAs did commonly start with uracil, there was no obvious preference for an adenine at position 10 (Figure S4c in Additional data file 3). Thus, while their other features do indicate their being some form of functional small RNA, these 26- and 27-U RNAs are some other kind of small RNA rather than piRNAs. However, the 28- and 29-U RNAs have a preference for an adenine at position 10 (Figure S4c in Additional data file 3); thus, they may be piRNA-like small RNAs.

### Different expression profiles of small RNAs in the two phases

Small RNAs in the gregarious library were enriched for lengths of 22-23 nucleotides, a typical length for animal miRNAs, and those in the solitary library were enriched for lengths of 26-29 nucleotides and 22-23 nucleotides (Figure 1a). For small RNAs shorter than 22 nucleotides, the gregarious locust has a higher expression level than the solitary locust, while for those longer than 22 nucleotides, the opposite is the case. In addition to the different length distributions of the small RNAs, the proportions of each type of small RNA in the libraries between the two phases were different (Figure 1b). The proportion of miRNAs in the gregarious phase is nearly two times as much as that in the solitary phase; however, endo-siRNAs and piRNA-like small RNAs make up a larger proportion in the solitary phase compared with those in the gregarious phase. There are more unannotated small RNAs in the solitary phase, indicating their potential functions, although we could not annotate them. In summary, the small RNA transcriptomes of the two phases show big differences in their length distribution and composition.

**Figure 7:**
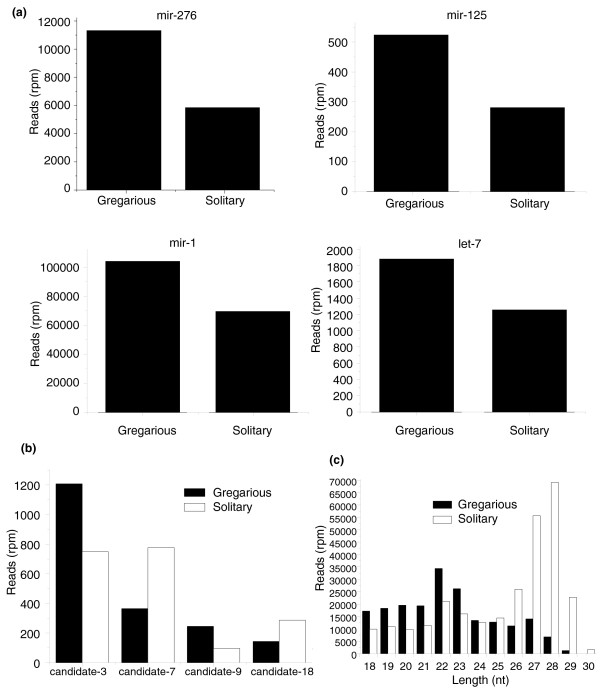
Different expression levels of small RNAs in the gregarious and solitary phases. **(a) **Four examples of conserved miRNAs that have distinct expression levels between the gregarious and solitary phases. **(b) **Four examples of locust-specific miRNAs that have distinct expression levels between the gregarious and solitary phases. **(c) **Different length distribution of the unannotated sequences in the two libraries. Rpm, reads per million.

We converted the reads of each kind of small RNA into reads per million (rpm) in order to make a comparison between the gregarious and solitary small RNA libraries. Almost each kind of small RNA, including miRNAs, endo-siRNAs, and piRNA-like small RNAs, had some differences in expression level between the two phases. Seventeen conserved and 84 predicted locust-specific miRNAs differed by ≥ 1.5-fold between the two phases. Four examples of conserved miRNAs (*mir-276*, *mir-125*, *mir-1*, and *let-7*) in the gregarious phase were shown to be expressed 1.94-fold, 1.87-fold, 1.5-fold, and 1.5-fold as much as in the solitary phase, respectively (Figure 7a). Also, some locust-specific miRNAs had a 1.5-24-fold difference in abundance between the two phases (Figure 7b).

Compared with those in the gregarious library, there are more abundant endo-siRNAs and piRNA-like small RNAs in the solitary library (Figure 6a; Tables S5 and S6 in Additional data file 3). For the endo-siRNAs shared in both libraries, only 3 in the gregarious phase were expressed at least 1.5-times as much as they were in the solitary phase. However, 26 endo-siRNAs in the solitary phase were expressed 1.67-54-fold as much as they were in the gregarious phase. Moreover, there are 86 solitary phase-specific siRNAs (≥ 5 reads), while there are only 6 gregarious phase-specific ones. We also observed that endo-siRNAs came from 2,307 unigenes of the ESTs, 319 of which generated siRNAs in both phases. However, 325 unigenes generated siRNAs only in the gregarious phase, and 1,663 only in the solitary phase. For piRNA-like small RNAs, the situation is similar to that of endo-siRNAs; 8 piRNA-like small RNAs differed more than 1.5-fold in abundance between the gregarious and solitary libraries, and only one of them was more abundant in the gregarious locust. There were no gregarious phase-specific piRNA-like small RNAs (≥ 5 reads), compared with 36 solitary phase-specific ones.

There were huge differences between the two phases in the expression levels of these unannotated sequences. Similar to those of all small RNAs, the expression levels of most of the longer (26-29 nucleotide) small RNAs in the solitary phase were much higher than in the gregarious phase (Figure 7c; Figure S4 in Additional data file 3), indicating a potential role of these 26-29 nucleotide small RNAs in the phase changes of locust.

## Discussion

### Evolution of conserved miRNAs

Nearly one-third of miRNAs from *D. melanogaster *in miRBase 11.0 [18] are conserved in the locust, indicating the bulk of miRNAs in the locust are composed of conserved and lineage-specific RNAs. Although some miRNAs are conserved in a wide range of species, our study shows that there are some species-specific nucleotide substitutions in the flanking regions of the 'seed' sequences in most of the conserved families. For example, the miR-190 sequences in the vertebrates we examined are the same (Figure 5b), but a different miR-190 sequence is found, and shared, in the insect species analyzed. In other words, the same miRNA family of closely related species can be clustered separately from that of other closely related species.

Focusing on these conserved substitutions, separate from the 'seed' sequence [6], it is apparent that some highly conserved miRNA families can also be regarded as 'species-specific' (Figure 5). The 'seed' sequence is important for mRNA target recognition, but it alone is not sufficient for miRNA-target interaction. Given such conserved substitutions, it is possible that these are present in parts of the mature miRNAs that are also involved in target recognition. Such findings provide a clue that miRNA target recognition may be a complex process.

**Figure 8:**
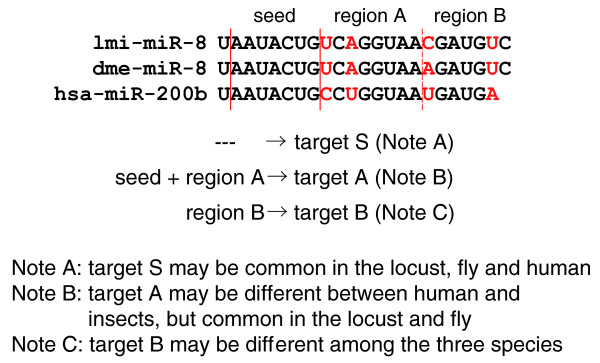
The same miRNA family plays roles in different modes in different species. Locust, fruit fly and human have the same targets if only the 'seed' sequence is used for target recognition. If the 'seed' and region A work together, mir-8 can regulate different targets in insects and human. If the 'seed' and region B work together, mir-8 in these three species can regulate species-specific targets. hsa-miR-200b belongs to the family of mir-8.

We propose that these conserved regions of sequence substitutions could be classified into different functional groups, which we call as A, B...and so on. The 'seed' plus region A, for example, may lead the miRNA to interact with target A, whereas 'seed' plus region B would lead to an alternative target repression, and so on (Figure 8). According to this model, we propose that nucleotide substitutions in conserved miRNAs would lead to the generation of new functions for corresponding miRNAs without changing their original functions. From an evolutionary view, using different modes of the same miRNAs to regulate the expression of multiple genes would be advantageous as it is both more economical and safer than producing completely new miRNAs, which would most likely cause more harm than good at a high cost upon their emergence [37]. In much the same way, protein evolution often occurs through changes in specific domains that become conserved during evolution as new functions arise with these domain changes. This hypothesis also explains why such sequence substitutions in the miRNA would be maintained in even distantly related species.

### Conservation of miRNA*

Previous studies have often ignored the function of miRNA* because these sequences are usually regarded as important primarily for maintaining the miRNA precursor secondary structure [1,24]. However, our research showed high sequence similarity for miRNA* between the locust and fruit fly (Figure 2) even though these two species diverged 350 million years ago [25]. This indicates that miRNA* may also play a functional role in some biological processes. A recent study [24] also indicated this may be the case, as they found miRNA* conservation among 12 sequenced Drosophilids. These 12 Drosophilids, however, were evolutionarily closely related. The findings in our study between the long separated locust and fruit fly provide even stronger support for a biologically functional role of miRNA* beyond maintenance of precursor secondary structure. Overall, with regards to miRNA* conservation, our findings indicate that organisms regulate mRNA expression in an economic way, using both miRNAs and miRNA*s.

### Reliability of the method to identify locust-specific miRNAs

We identified non-conserved miRNA families in the locust due to the application of a new method we developed based on the biogenesis features of miRNAs [16,20,38], which is independent of available genome sequence data. To estimate the validity of the predicted miRNA candidates, we used a PCR-based method to determine their locations in the locust genome on the basis of the secondary structure features of their precursors. We were able to validate 13 of our chosen 24 predicted miRNAs by our PCR-based method and determined that their sequences in conjunction with their flanking sequences in the genome could be folded into perfect miRNA-like hairpin structures. These results provide strong support for the reliability of our method to predict species-specific miRNAs. Although 11 of the chosen predicted miRNAs did not provide positive results, we felt that this was likely due to the low quality of primers we used, as we could only refer to short sequences during primer design. Given these limitations, we estimated that our false positive prediction rate would actually be lower than 40%. Overall, the principles we adopted in this study were stringent, especially in our allowance for mismatches (only four) between the predicted miRNA duplex-like pairs. In reality, some canonical miRNAs have more than four mismatches with their star sequences [18]. So if less stringent mismatch criteria were used, it is likely that more miRNAs could be found (data not shown). This, however, would also be accompanied with a higher false positive rate due to allowed base-paring of random, rather than true, miRNA sequences. Thus, in this study, to minimize the false positive rate, we chose to use the more stringent mismatch limits when we searched for miRNA duplex-like pairs in the library.

In addition to the PCR-based method of validation, we also assessed the reliability of our miRNA prediction method by using *Drosophila *miRNA data (see Methods in Additional data file 3). The findings here provide additional support for the feasibility and reliability of our method. We believe that more than 100 of our predicted locust-specific miRNAs would be canonical.

Although the principle of our method, based on features of miRNA biogenesis, coincides with that of miRDeep [39], our method is more effective for finding miRNA duplex-like pairs when there is little available genome sequence information. Therefore, our method could be used to identify miRNAs in a wider variety of organisms, particularly those without whole genome sequence data. Additionally, we have also provided a simple experimental method to validate the reliability of the results predicted by our computational method. Combining our computational approaches with experimental methods, novel and non-conserved miRNAs can be identified from any species regardless of the absence of their genome sequence data. Being able to identify more novel miRNAs in a greater number of species even in the absence of genome sequence data will be invaluable in improving our understanding of the evolution and function of miRNAs.

### Target prediction of miRNAs

It is difficult to predict miRNA targets in animals because the detailed mechanism of interaction between miRNA and its target transcripts is not clear, although several bioinformatic tools have been developed, such as miRanda. None of the available computational methods can predict miRNA targets accurately and they all give results with higher false positive rates [20,32,35]. Moreover, most miRNAs target 3' UTR sequences of mRNAs in animals, so it is more difficult to predict targets of locust miRNAs without a complete 3' UTR database. Alternatively, we chose to use a locust EST database to predict the targets of its miRNAs. Although it is possible to find some canonical targets of miRNAs, using the EST database will lead to higher false positive rates compared with 3' UTR database when predicting targets using bioinformatic tools. Combining the two factors above, although we predicted several targets of locust miRNAs using miRanda, it is very likely that there are some false positives.

We found that some mRNAs expressed differently between the two phases of the locust were potential targets of miRNAs. Because gene expression can be regulated at both the transcriptional and post-transcriptional levels, we believe it is possible that post-transcriptional gene expression regulation contributes to differential expression of genes between the two phases of the locust, such as the locust *pale *gene, a potential target of lmi-miR-133 (Figure 4c). However, validation of the relationship between miRNAs and mRNA transcripts expressed differentially between the two phases needs more experimental evidence.

### The scope of small RNAs in the locust

High-throughput sequencing of small RNAs showed that there were a large number of small RNAs in the locust transcriptome (Figure 1). Our results indicate that there are fewer miRNAs in insects than in mammals; this is likely because there was an expansion in the number of miRNAs at the advent of vertebrates and mammals [40]. We did find evidence for the existence of several different kinds of small RNAs in the locust, although the proportion of endo-siRNAs and piRNA-like small RNAs identified in the locust was small. We expect that more endo-siRNAs and piRNAs will be identified with an increase in available locust genome and transcriptome data.

A global survey of small RNAs in the locust would contribute additional information to understanding the function and evolution of small RNAs in insects. The analysis of the characteristics and expression of small RNAs in the locust enhances the knowledge of gene expression regulation on non-model and hemimetabolous insects at the post-transcriptional level and enables comparison of long-term evolutionary history between the homo- and hemimetabolous insects.

The locust has already been used in a variety of ways as a good model for understanding the mechanisms of the immune system and neural pathways [12,13], in both of which small RNA gene regulation systems might be involved [5,11]. Moreover, many of the small RNAs of the locust, a typical hemimetabolous insect, likely have important functions in complex developmental processes.

### Small RNAs involved in phase changes

Significant differences in small RNA expression levels between the gregarious and the solitary phases indicate their potential functions in phase transition of the locust. The two phases of the locust share the same genome but exhibit different gene expression profiles and phenotypes, suggesting different regulation of gene expression [13]. Comparison between the coding genes of the two phases at the expression level has been done and many interesting genes have been found to be involved in phase changes of the locust [13,41]. Also, our study shows that the expression patterns of non-coding RNAs differ between the gregarious and the solitary phases, suggesting that phenotypic differences between the two phases are epigenetic changes but not derived from genomic differences.

We found 17 conserved miRNAs to have different expression levels between the gregarious and solitary phases. These miRNAs may be involved in gene expression regulation at the post-transcriptional level during phase transition. Especially, we are most interested in 5 of the 17 miRNAs that are expressed differentially in the two phases. *mir-276 *has the biggest expression difference between the two phases (Figure 7a), although there has been no reports about its function. Such a difference might imply its functional role in the phase transition of the locust. *let-7 *and *mir-125 *regulate metamorphic processes in *C. elegans *and *Drosophila *[42,43]. Phase changes in locusts can only happen before they have become adults; solitary locusts can only swarm during the larval stages and no once they have reached the adult stage. Since the two miRNAs (*let-7 *and *mir-125*) and the phenomena of phase changes are both linked with metamorphic processes, we think that the two miRNAs and the phenotype of phase changes may be related. *mir-1 *and *mir-315 *also have different expression levels (data on *mir-315 *expression levels is not shown). *mir-1 *is a muscle-specific miRNA [28], and *mir-315 *is a potent activator of Wingless signaling in *Drosophila *[44]. Because it is related to the thorax muscle and the wing, we think that the difference in flying ability between gregarious and solitary locusts may be regulated by *mir-1 *and *mir-315*.

Based on our analysis of the small RNA expression levels in gregarious and solitary locusts, we believe that some small RNAs that regulate the expression of protein coding genes in the two phases must be involved in the process of phase changes. It is possible that we could provide insight into the phase changes and find new approaches to control the locust plagues throughout the world by small RNAs.

## Conclusion

High-throughput sequencing provides a good chance for us to study small RNAs in the locust, which is an important worldwide pest. This study led to the discovery of a large number of small RNAs in the locust, including miRNAs, endo-siRNAs and piRNA-like small RNAs. Importantly, we have identified 185 potential locust-specific miRNA candidates using the method we developed, although there is no locust genome sequence available. Our method makes it possible to discover more miRNA families in a broader range of species whose genome sequences have not been sequenced. We further show the evolutionary path of miRNAs in the locust, indicating the potential evolutionary mechanism of miRNAs. The function of small RNAs in phase changes of the locust is disclosed in our study. We found significant differences in the expression of small RNAs between the two phases of the locust and target prediction shows that some genes expressed differentially in the two phases are targets of miRNAs, which gives us clues to further discover the mechanisms of phase change in locusts.

## Materials and methods

### Preparation of total RNA

Total RNA was extracted using TRIzol reagent (Invitrogen, Carlsbad, CA, USA) from mixed-stage (including embryos, every instar larvae and adults) *Locusta migratoria *that we fed in our lab. We collected 0-1, 2-3, 4-5, 6-7, 8-9, 10-11, 12-13 and 14-15 day-old embryos cultured at 30°C in clean sand with relative humidity. For the larvae, we collected the whole body except the midgut and pooled them to ensure every instar was present in the sample. We chose to collect adults at eclosion, sexual maturation, post-spawning, and elderly stages separately and then pooled them together. Total RNA was extracted according to the manufacturer's protocol. We examined the quality of RNA using an Agilent 2100 Bioanalyzer.

### Small RNA library construction and high-throughput sequencing

RNA fragments 14-30 bases long were isolated from total RNA by Novex 15% TBE-Urea gel (Invitrogen). Then, a 5' adaptor (Illumina, San Diego, CA, USA) was ligated to purified small RNAs followed by purification of ligation products on Novex 15% TBE-Urea gel. The 5' ligation products were then ligated to a 3' adaptor (Illumina) and products with 5' and 3' adaptors were purified from Novex 10% TBE-Urea gel (Invitrogen). Subsequently, these ligation products were reverse transcribed followed by PCR amplification. The amplification products were excised from 6% TBE-Urea gel (Invitrogen). The purified DNA fragments were used for clustering and sequencing by Illumina Genome Analyzer at the Beijing Genomics Institute, Shenzhen.

### Discovery of conserved locust miRNA families

We discarded bad reads that were the result of incorrect sequencing or were the reads of adaptor contamination that were not ligated to any other sequences. We clustered the remaining reads based on sequence similarity and the dominant reads were analyzed as follows: the reads were analyzed by BLAST against EST database [13] and FlyBase [17] to discard rRNA, tRNA and snRNA. Subsequently, the remaining sequences were analyzed by BLAST search against miRBase v11.0 [18]. Sequences in our libraries with identical or related (four or fewer nucleotide substitutions) sequences from *D. melanogaster *or other insects (mosquito, silkworm, and honeybee) were identified as conserved miRNAs.

### Discovery of non-conserved locust miRNA families

We first looked at the high-throughput sequencing data of small RNAs in other species, including *C. elegans*, *D. melanogaster*, and *Arabidopsis *[16,20,38], and found that star sequences of most miRNAs were also present in the small RNA libraries, and that the miRNA-miRNA* duplexes exhibited 1 or 2 nucleotide 3' overhangs, a characteristic of RNase III enzyme cleavage (Figure 3a). The 5' end sequences of miRNA clusters showed obvious consistency compared with other small RNAs and degradation fragments (Figure 3a). Thus, if a sequence in the locust small RNA libraries is a canonical miRNA, its star sequence should be identified based on imperfect base-pairing and a 1-2 nucleotide 3' overhang when paired with its complementary mature miRNA.

Based on the biogenesis features of miRNA (Figure 3a), we developed a perl script to search for possible candidate miRNA-miRNA* duplexes, which satisfied the following criteria: they were selected primarily by base-pairing, allowing for G:U pairing, which is common in the miRNA precursors; they could contain up to four mismatches; they could have a maximum size of 4 nucleotides for a bulge in the candidate miRNA sequence; they had to have a 1-2 nucleotide 3' overhang [45,46]; the dominant strand had to have five or more reads in the library because miRNAs with a low expression level were likely to have no star form in the library; the length of the dominant strand had to be between 18 and 24 nuceotides long; the 5' ends in more than 80% of the reads of those sequences in the cluster of the dominant sequence of the pairs had to be consistent with each other. After these criteria were met, we then used mfold to evaluate the ability of the identified pairs to form a hairpin structure [22,23], where their free energy of folding (ΔG) was an important standard for use in determining the stability of RNA secondary structure.

In order to satisfy the requirement of input sequences analyzed by mfold, we joined the two sequences in each candidate pair using a standard hairpin-forming linker sequence (GCGGGGACGC). Those pairs that met the following conditions were analyzed further: the pairs had a free energy less than or equal to -21 kcal/mol (in cases where there was more than one partner for a sequence, the pair with the lowest free energy was selected as the true one); the pairs had no bulge bigger than 6 nucleotides and multiple loops. The way of determining the best parameters and of testing this method is described in the Methods in Additional data file 3.

### Amplification of the miRNA precursors from locust genomic DNA

We extracted genomic DNA from the fifth instar locust using a Gentra Puregene Tissue Kit (Qiagen, Valencia, CA, USA) according to the manufacture's protocol. We designed primers for 8 conserved miRNAs and 24 candidate miRNA-miRNA* pairs we predicted based on a dependence of the sequences of the mature miRNA and miRNA* species using Primer Premier 5.0. (Premier Biosoft International, Palo Alto, CA, USA) Because mature miRNA may come from either arm of the precursor, we designed two pairs of primers for each duplex. Corresponding fragments were amplified by PCR and the length of amplification products was examined on 2.5% agarose gels. Fragments between 55 and 70 nucleotides in length were subcloned into pMD18-T vector (Takara, Dalian, Liaoning, China) for sequencing analyses.

### Discovery of endo-siRNAs and piRNA-like small RNAs using ESTs

The 23-29 nucleotide long RNAs matching ESTs annotated as transposons were considered as piRNA-like small RNAs. Those small RNAs that perfectly matched EST antisense strands were considered as candidate endo-siRNAs if they were not from annotated transposons. Moreover, we also searched the ESTs for miRNA precursors. Although there were some sequences that perfectly matched EST sense strands, no typical hairpin structure of these ESTs could be identified using mfold. Rather than folding the entire EST sequence, regions of 70 nucleotides, 100 nucleotides and 150 nucleotides on either side of the small RNA sequences were folded.

### Prediction of miRNA targets

Unigene sequences from the EST database of the locust [13,14] were chosen to predict the miRNA targets without distinguishing the 3' UTR from the protein coding region. miRanda v3.1 [35] was selected as the prediction tool. A miRanda score greater than 150 was used to select unigene targets.

### 3' RACE of the locust pale gene

The 3' UTR sequence of the locust *pale *gene was obtained by 3' rapid amplification of cDNA ends (RACE) using a SMART RACE cDNA Amplification Kit (Clontech, Takara, Dalian, Liaoning, China) with the primer GCGACCTGGACAACTGCAACCACCTCAT according to the manufacturer's protocol.

## Abbreviations

endo-siRNA: endogenous siRNA; EST: expressed sequence tag; lmi-miR number: locust miR-number; miRNA: microRNA; piRNA: piwi-associated RNA; RACE: rapid amplification of cDNA ends; siRNA: small interfering RNA; UTR: untranslated region.

## Authors' contributions

YW and LK designed the study. YW performed all the bioinformatic analysis except target prediction of miRNAs. SC provided help to analyze piRNA-like small RNAs. PY predicted targets of miRNAs and YW analyzed the prediction results. SC and YW isolated total RNA and carried out PCR experiments. ZM carried out 3' RACE experiments. YW and LK prepared the manuscript. All authors read and approved the final manuscript.

## Additional data files

The following additional data are available with the online version of this paper. Additional data file 1 lists sequences of locust endo-siRNAs. Additional data file 2 lists sequences of locust piRNA-like small RNAs. Additional data file 3 contains four supplemental figures (Figures S1-S4), six supplemental tables (Tables S1-S6), and supplemental methods. Figure S1 shows alignment of miR-79 and miR-10 of different species. Figure S2 shows expression patterns of locust miRNAs. Figure S3 shows transposon types from which small RNAs are derived in the locust. Figure S4 shows lengths and initial nucleotide distributions of the unannotated small RNA sequences. Table S1 lists the sequences of conserved miRNAs and miRNA*s in the locust. Table S2 lists precursor sequences of the seven conserved miRNAs that have a conserved star sequence. Table S3 lists sequences of predicted locust-specific miRNAs. Table S4 lists the ten most abundant miRNA-like 5'-end small RNAs in the remaining reads after annotation of miRNAs, siRNAs and piRNA-like small RNAs. Table S5 lists endo-siRNAs with different expression levels between the two phases. Table S6 lists piRNA-like small RNAs with different expression levels between the two phases. The Methods show the way to determine the best parameters of our miRNA prediction method and assess the reliability of our method using *Drosophila *miRNA data.

## Supplementary Material

Additional data file 1 Locust endo-siRNAs. Click here for file

Additional data file 2 Locust piRNA-like small RNAs. Click here for file

Additional data file 3 Figure S1 shows alignment of miR-79 and miR-10 of different species. Figure S2 shows expression patterns of locust miRNAs. Figure S3 shows transposon types from which small RNAs are derived in the locust. Figure S4 shows lengths and initial nucleotide distributions of the unannotated small RNA sequences. Table S1 lists the sequences of conserved miRNAs and miRNA*s in the locust. Table S2 lists precursor sequences of the seven conserved miRNAs that have a conserved star sequence. Table S3 lists sequences of predicted locust-specific miRNAs. Table S4 lists the ten most abundant miRNA-like 5'-end small RNAs in the remaining reads after annotation of miRNAs, siRNAs and piRNA-like small RNAs. Table S5 lists endo-siRNAs with different expression levels between the two phases. Table S6 lists piRNA-like small RNAs with different expression levels between the two phases. The Methods show the way to determine the best parameters of our miRNA prediction method and assess the reliability of our method using *Drosophila *miRNA data. Click here for file
