# Supplementary material for: Characterization and comparative profiling of the small RNA transcriptomes in two phases of locust
Source: Genome Biol. 2009 Jan 16;10(1):R6. doi: 10.1186/gb-2009-10-1-r6 (PMC2687794; doi:10.1186/gb-2009-10-1-r6)
Supplement: Additional data file 3 — Figure S1 shows alignment of miR-79 and miR-10 of different species. Figure S2 shows expression patterns of locust miRNAs. Figure S3 shows transposon types from which small RNAs are derived in the locust. Figure S4 shows lengths and initial nucleotide distributions of the unannotated small RNA sequences. Table S1 lists the sequences of conserved miRNAs and miRNA*s in the locust. Table S2 lists precursor sequences of the seven conserved miRNAs that have a conserved star sequence. Table S3 lists sequences of predicted locust-specific miRNAs. Table S4 lists the ten most abundant miRNA-like 5'-end small RNAs in the remaining reads after annotation of miRNAs, siRNAs and piRNA-like small RNAs. Table S5 lists endo-siRNAs with different expression levels between the two phases. Table S6 lists piRNA-like small RNAs with different expression levels between the two phases. The Methods show the way to determine the best parameters of our miRNA prediction method and assess the reliability of our method using Drosophila miRNA data. [file gb-2009-10-1-r6-S3.pdf]

Figure S1 - Alignment of miR-79 and miR-10 of different species.

The presence of an additional nucleotide, highlighted by red color, at the 5' end of the locust miRNA creates a different 'seed' sequence (2-8 nucleotides) compared to other insects.

lmi: *L. migratoria*; cel: *C. elegans*; cbr: *C. briggsae*; dme: *D. melanogaster*; dps: *D. pseudoobscura*; aga: *A. gambiae*; ame: *A. mellifera*; hsa: *H. sapiens*; mmu: *M. musculus*; dre: *D. rerio*; xtr: *X. tropicalis*; bmo: *B. mori*; gga: *G. gallus*.

Figure S2 - Expression patterns of locust miRNAs. The bars show reads of miRNAs.

Figure S3 - Transposon types deriving small RNAs. The bars show reads of small RNAs from the corresponding transposons.

Figure S4 - Length and initial nucleotide distribution of the unannotated small RNA sequences.

(A) Length and initial nucleotide distribution of the unannotated reads in the gregarious library; (B) Length and initial nucleotide distribution of the unannotated reads in the solitary library; (C) Nucleotide distribution at position 10 of the unannotated 26-, 27-, 28-, and 29-mer RNAs in the locust.

Figure S1.

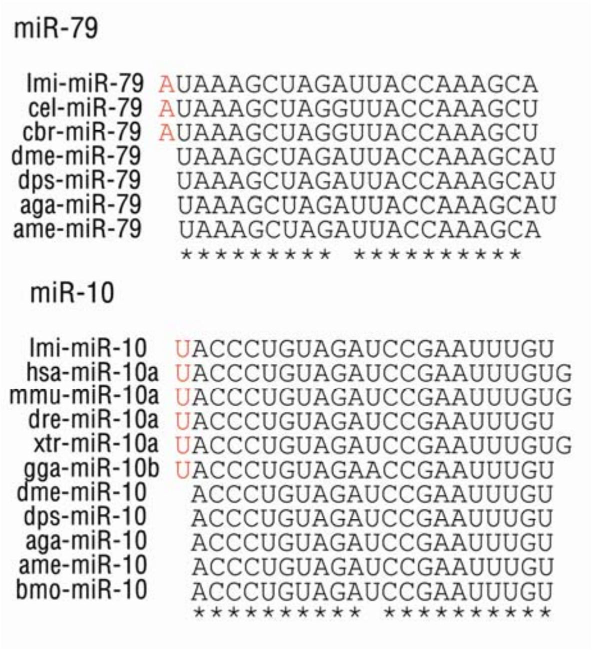

miR-79
lmi-miR-79 AUAAAGCUAGAUUACCAAAGCA
cel-miR-79 AUAAAGCUAGGUUACCAAAGCU
cbr-miR-79 AUAAAGCUAGGUUACCAAAGCU
dme-miR-79 UAAAGCUAGAUUACCAAAGCAU
dps-miR-79 UAAAGCUAGAUUACCAAAGCAU
aga-miR-79 UAAAGCUAGAUUACCAAAGCAU
ame-miR-79 UAAAGCUAGAUUACCAAAGCA
********* **********
miR-10
lmi-miR-10 UACCCUGUAGAUCCGAAUUUGU
hsa-miR-10a UACCCUGUAGAUCCGAAUUUGUG
mmu-miR-10a UACCCUGUAGAUCCGAAUUUGUG
dre-miR-10a UACCCUGUAGAUCCGAAUUUGU
xtr-miR-10a UACCCUGUAGAUCCGAAUUUGUG
gga-miR-10b UACCCUGUAGAACCGAAUUUGU
dme-miR-10 ACCCUGUAGAUCCGAAUUUGU
dps-miR-10 ACCCUGUAGAUCCGAAUUUGU
aga-miR-10 ACCCUGUAGAUCCGAAUUUGU
ame-miR-10 ACCCUGUAGAUCCGAAUUUGU
bmo-miR-10 ACCCUGUAGAUCCGAAUUUGU
********** **********

Figure S2.

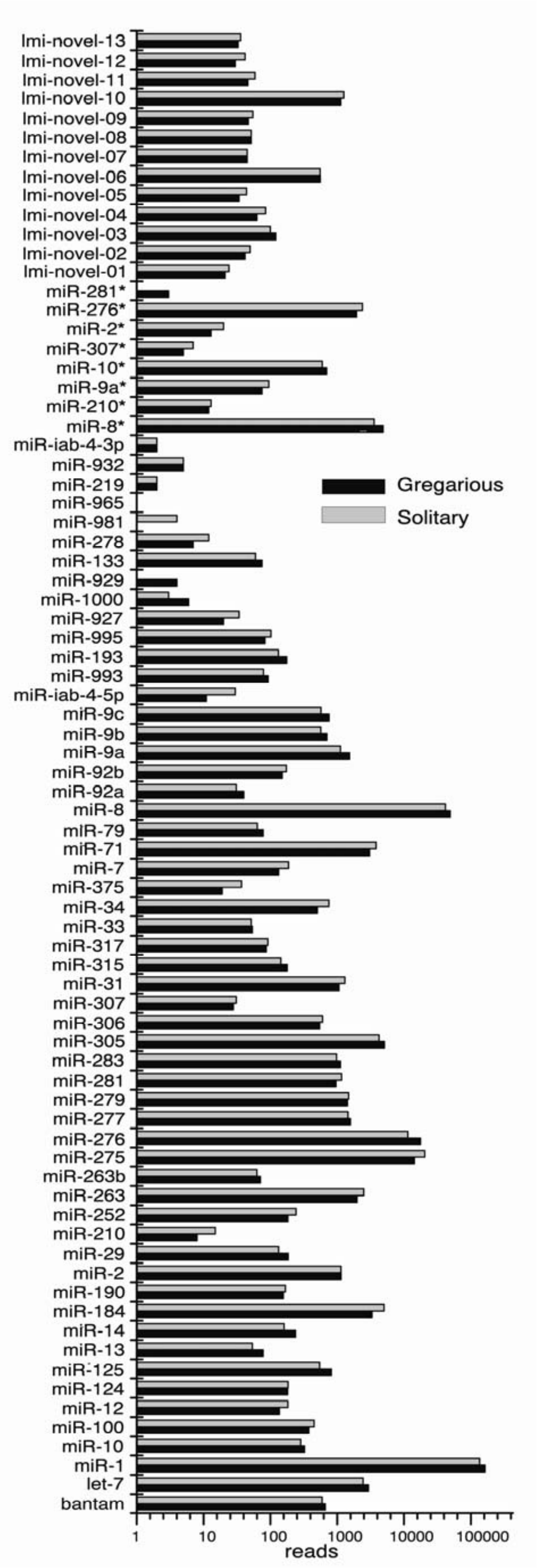
lmi-novel-13
lmi-novel-12
lmi-novel-11
lmi-novel-10
lmi-novel-09
lmi-novel-08
lmi-novel-07
lmi-novel-06
lmi-novel-05
lmi-novel-04
lmi-novel-03
lmi-novel-02
lmi-novel-01
miR-281*
miR-276*
miR-2*
miR-307*
miR-10*
miR-9a*
miR-210*
miR-8*
miR-iab-4-3p
miR-932
miR-219
miR-965
miR-981
miR-278
miR-133
miR-929
miR-1000
miR-927
miR-995
miR-193
miR-993
miR-iab-4-5p
miR-9c
miR-9b
miR-9a
miR-92b
miR-92a
miR-8
miR-79
miR-71
miR-7
miR-375
miR-34
miR-33
miR-317
miR-315
miR-31
miR-307
miR-306
miR-305
miR-283
miR-281
miR-279
miR-277
miR-276
miR-275
miR-263b
miR-263
miR-252
miR-210
miR-29
miR-2
miR-190
miR-184
miR-14
miR-13
miR-125
miR-124
miR-12
miR-100
miR-10
miR-1
let-7
bantam
Gregarious
Solitary
1
10
100
1000
10000
100000
reads

Figure S3.

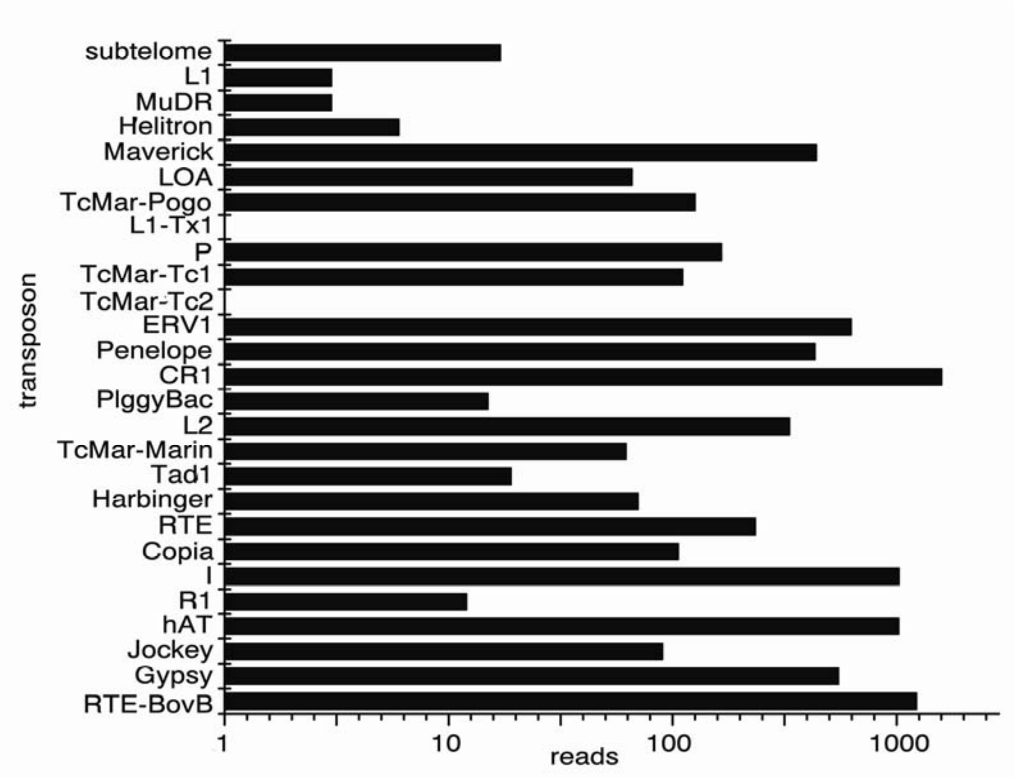

subtelome
L1
MuDR
Helitron
Maverick
LOA
TcMar-Pogo
L1-Tx1
P
TcMar-Tc1
TcMar-Tc2
ERV1
Penelope
CR1
PiggyBac
L2
TcMar-Marin
Tad1
Harbinger
RTE
Copia
I
R1
hAT
Jockey
Gypsy
RTE-BovB
transposon
1
10
100
1000
reads

Figure S4.

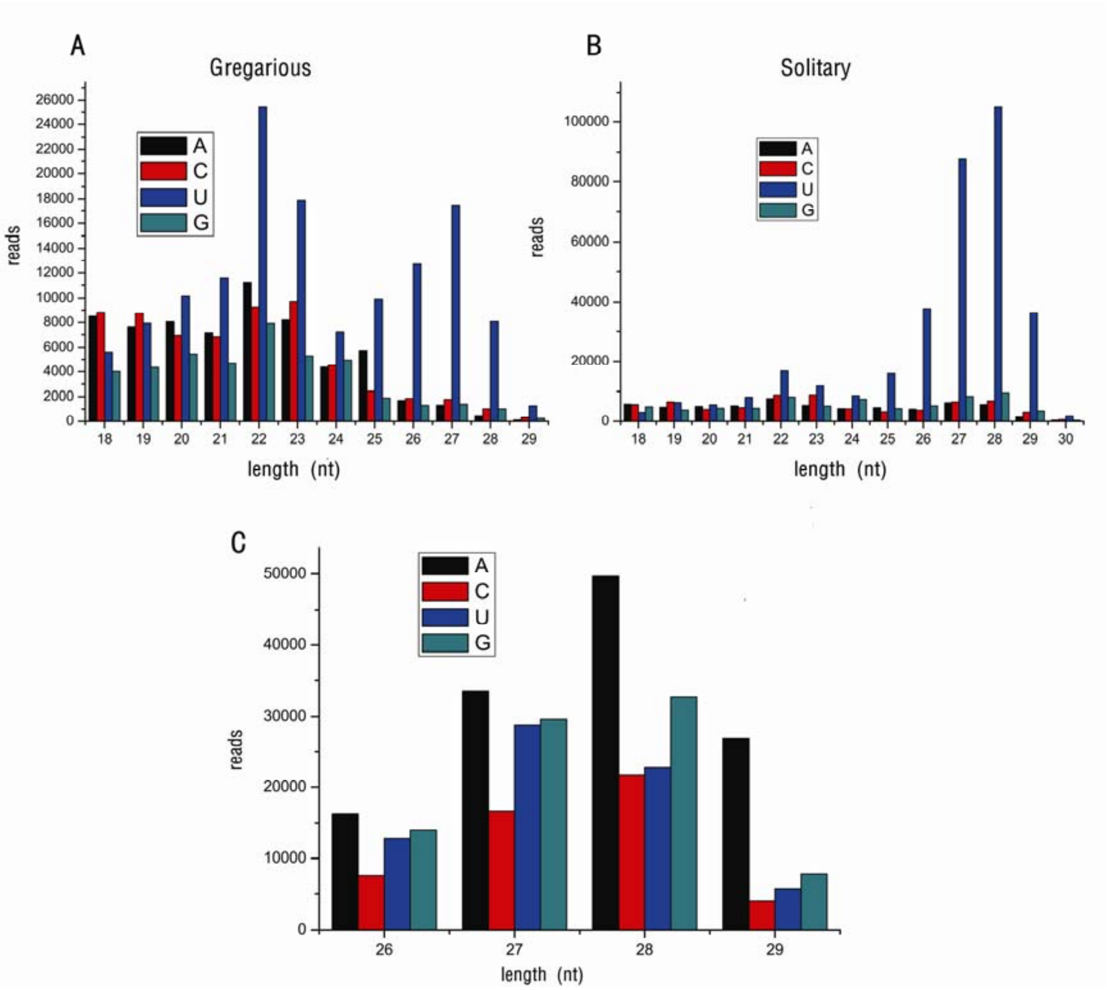

Table S1. Conserved miRNAs and miRNA*s in the locust.

miRNA name	Sequence in the locust (5'-3')	miRNA family
lmi-bantam	UGAGAUCAUUGUGAAAGCUGAUU	bantam
lmi-let-7	UGAGGUAGUAGGUUGUAUAGUU	let-7
lmi-miR-1	UGGAAUGUAAAGAAGUAUGGAG	miR-1
lmi-miR-2	UAUCACAGCCAGCUUUGAUGA	miR-2
lmi-miR-7	UGGAAGACUAGUGAUUUUGUUGU	miR-7
lmi-miR-8	UAAUACUGUCAGGUAACGAUGUC	miR-8
lmi-miR-9a	UCUUUGGUUAUCUAGCUGUAUGA	miR-9
lmi-miR-9b	UCUUUGGUGAUCUAGCUG	miR-9
lmi-miR-9c	UCUUUGGUAUCCUAGCUGU	miR-9
lmi-miR-10	UACCCUGUAGAUCCGAAUUUGU	miR-10
lmi-miR-12	UGAGUAUUACAUCAGGUACU	miR-12
lmi-miR-13	UAUCACAGCCACUUUGAUGA	miR-2
lmi-miR-14	UCAGUCUUUUUCUCUCUCCUAU	miR-14
lmi-miR-29	UAGCACCAUUUGAAAUCAGU	miR-29
lmi-miR-31	AGGCAAGAUGUCGGCAUAGCU	miR-31
lmi-miR-33	GUGCAUUGUAGUUGCAUUGCA	miR-33
lmi-miR-34	UGGCAGUGUGGUUAGCUGGUU	miR-34
lmi-miR-71	UGAAAGACAUGGGUAGUGAGAU	miR-71
lmi-miR-79	AUAAAGCUAGAUUACCAAAGCA	
lmi-miR-92a	UAUUGCACUUGUCCCGGCCUAU	miR-25
lmi-miR-92b	AAUUGCACUUUUCCCGGCCUG	miR-25
lmi-miR-100	AACCCGUAGAUCCGAACUUGUG	miR-100
lmi-miR-124	UAAGGCACGCGGUGAAUGCCAA	miR-124
lmi-miR-125	UCCCUGAGACCCUAACUUGUGA	miR-125
lmi-miR-133	UUGGUCCCCUUCAACCAGCUGU	miR-133
lmi-miR-184	UGGACGGAGAACUGAUAAGGGC	miR-184
lmi-miR-190	AGAUAUGUUUGAUAUUCUUGGUUG	miR-190
lmi-miR-210	CUUGUGCGUGUGACAGCGGCUAU	miR-210
lmi-miR-219	UGAUUGUCCAAACGCAAUUCUUGA	miR-219
lmi-miR-263a	AAUGGCACUGGAAGAAUUCACGG	miR-263
lmi-miR-263b	CUUGGCACUGGAAGAAUUCACAGA	miR-263
lmi-miR-275	UCAGGUACCUGAAGUAGCGCGCG	miR-275
lmi-miR-276	UAGGAACUUCAUACCGUGCUCU	miR-276
lmi-miR-277	UAAAUGCACUAUCUGGUACGACA	miR-277
lmi-miR-278	UCGGUGGGACUUUCGUCCGUUU	miR-278
lmi-miR-279	UGACUAGAUCCAUACUCGU	miR-279
lmi-miR-281	UGUCAUGGAGUUGCUCUCUUUAA	miR-46
lmi-miR-283	AAAUAUCAGUUGGUAAUUCU	miR-283
lmi-miR-305	AUUGUACUUCAUCAGGUG	miR-305
lmi-miR-306	UCAGGUACUGAGUGACUCUGA	miR-306

lmi-miR-307	UCACAACCUCCUUGAGUGAGCGA	miR-67
lmi-miR-315	UUUUGAUUGUUGCUCAGAAAGC	miR-315
lmi-miR-317	UGAACACAGCUGGUGGUAUCU	miR-317
lmi-miR-375	UUUGUUCGCUCGGCUCGAG	miR-375
lmi-miR-iab-4-5p	ACGUAUACUGAAUGUAUCCUGA	miR-iab
lmi-miR-iab-4-3p	CGGUAUACCUUCAGUAUACGUA	miR-iab
lmi-miR-252	CUAAGUACUAGUGCCGCAGGAG	
lmi-miR-193	UACUGGCCUGCUAAGUCCCAA	
lmi-miR-995	UAGCACCACAUGAUUCAGCUUA	
lmi-miR-927	UUUAGAAUUCCUACGCUUUACC	miR-927
lmi-miR-1000	AUAUUGUCCUGUCACAGCAGUA	
lmi-miR-929	CUCCCUAACGGAGUCAGGUUGU	miR-929
lmi-miR-932	UCAAUUCCGUAGUGCAUUGCAGU	
lmi-miR-965	UAAGCGUATAGCUUUUCCCCUUU	
lmi-miR-981	UUCGUUGUCGACGAAACCUGCA	
lmi-miR-993	GAAGCUCGUCUCUACAGGUAUCU	
lmi-miR-8*	CAUCUUACCGGGCAGCAUUAGA	
lmi-miR-9a*	AUAAAGCUAGGUUACCGAAGUUA	
lmi-miR-10*	AAAUUCGGUUCUAGAGAGGUUU	
lmi-miR-307*	ACUCACUCAACCUGGGUGUGAUG	
lmi-miR-210*	AGCUGCUGGACACUGCACAAGA	
lmi-miR-276*	AGCGAGGUAUAGAGUUCCUACG	
lmi-miR-281*	AAGAGAGCUAUCCGUCGACAGU	

Table S2. Precursor sequences of the seven conserved miRNAs holding conserved stars.

name	Sequences of the precursors (5’-3’)
mir-307	ACUCACUCAACCUGGGUGUGAUGUCCGUUGAGAGCCCGUCACAACCUCCUUGAGUGAGCGA
mir-276	UAGCGAGGUAUAGAGUUCCUACGUGUGUUGUUAUAGUAGGAACUUCAUACCGUGCUCU
mir-281	AAGAGAGCUAUCCGUCGACAGUACAGUGAGUAAACUGUCAUGGAGUUGCUCUCUUUAA
mir-210	AGCUGCUGGACACUGCACAAGAUUAGCGAUUAAAAGACUCUUGUGCGUGUGACAGCGGCUAU
mir-10	UACCCUGUAGAUCCGAAUUUGUUUGACAUAGUGCGACAAAUUCGGUUCUAGAGAGGUUU
mir-9a	UCUUUGGUUAUCUAGCUGUAUGAGUGCAUGUGUCUGUCAUAAAGCUAGGUUACCGAAGUUA
mir-8	AUCUUACCGGGCAGCAUUAGAUUGCGACUGAGAUUUCUAAUACUGUCAGGUAACGAUGUC

Table S3. Locust-specific miRNAs we predicted.

Name	Sequence (5'-3')	length
lmi-miR-candidate-1	UCACCCAGGACGGUGGAUCA	20
lmi-miR-candidate-2	UGGACGGAGAUAUGAUAAGGUC	22
lmi-miR-candidate-3	ACUAUUCUGUUAGUGACACUGAU	23
lmi-miR-candidate-4	GUAGGCCGGCGGAAACUACUUG	22
lmi-miR-candidate-5	CAAUUCAGGAUGUCUUGACCA	21
lmi-miR-candidate-6	UGGUAACUCCACCACCGUUGGCA	23
lmi-miR-candidate-7	UCUAAAGGAUUUGUUACAGACUG	23
lmi-miR-candidate-8	AGUUGGAAGUGGGGAUCUCGGCA	23
lmi-miR-candidate-9	AAGCACAUUUCUGACACUGUCA	22
lmi-miR-candidate-10	UUAGCAGAAGGUAAGUGACUCG	22
lmi-miR-candidate-11	AGACUCAAGUUCUGGUCCUC	20
lmi-miR-candidate-12	UGACUGGGUGAUGCAACAGUUG	22
lmi-miR-candidate-13	UGGACGGAGAUAUGAUAAGGUA	22
lmi-miR-candidate-14	UCUGAAGGAUUUGUUACAGACUGU	24
lmi-miR-candidate-15	AGCCAGUGCUUGGUGGAUCUGCU	23
lmi-miR-candidate-16	AAGAUUGCAGGUUCGGGUCC	20
lmi-miR-candidate-17	CACGUGACUCACCUACCGGAUA	22
lmi-miR-candidate-18	AGUUCAAUACUGGAGUAGACGCAA	24
lmi-miR-candidate-19	AAGCAUAUUCCUGAUACAGACA	22
lmi-miR-candidate-20	AUGGUGUCAGGAAUAUGAGUCG	22
lmi-miR-candidate-21	UCUUCGUCGCGGUUUGCUGCCAU	23
lmi-miR-candidate-22	UCUGGUAUGUGGGUCACUUGCAU	23
lmi-miR-candidate-23	UCCUGUUGCACCUGUUGGUUGAU	23
lmi-miR-candidate-24	UGGUUGCAUGCUUAUGACGUCA	22
lmi-miR-candidate-25	AGACAUAUUCCUGAUACUGACA	22
lmi-miR-candidate-26	UUUAGAAUUUGUACGCUUUGUU	22
lmi-miR-candidate-27	CAUCACAUUUGUAUGGUCUCAU	22
lmi-miR-candidate-28	UGUCUACCUCCACUGAUCCCGCU	23
lmi-miR-candidate-29	UAAGCUCGUCUUUCUGAGCAGU	22
lmi-miR-candidate-30	UGAUGUGCGUUUGAUGGAAACAG	23
lmi-miR-candidate-31	UGGGACGUUUUAGCAAAGGGCA	22
lmi-miR-candidate-32	UGUGCCUGUCGCAUCUCGUCGCU	23
lmi-miR-candidate-33	UGGUGAGUGGGAGCUGGGUAGCA	23
lmi-miR-candidate-34	CGGCACCUGUUGGAGUGCAAUUG	23
lmi-miR-candidate-35	UUUGUACCAGGAAUAUGAAUCG	22
lmi-miR-candidate-36	AAGCACAUUUCUGACACUGUCU	22
lmi-miR-candidate-37	UGACUGGGUGAUGCAUCAGUUG	22
lmi-miR-candidate-38	GGGUCGGUGGUGUAGGGGUAUC	22
lmi-miR-candidate-39	UAGCACCACAUGAUUCAGCUUA	22
lmi-miR-candidate-40	CAGGUACAAAUGCGCCGACCGC	22

lmi-miR-candidate-41	AGGGAGGUCUGAUGCCAUGU	20
lmi-miR-candidate-42	AAUUGCAGCCCAGGUACUCUCA	22
lmi-miR-candidate-43	UUAUGGUAGAGUGGUGACAACA	22
lmi-miR-candidate-44	AGGGAUAACUGGCUUGUGGCG	21
lmi-miR-candidate-45	CCUUGCUUGUGGGAUGAAGGUG	22
lmi-miR-candidate-46	ACAGGCUAGAAUCUCAGGUAU	21
lmi-miR-candidate-47	UAAUCUCAUGUGGUAACUGUGA	22
lmi-miR-candidate-48	GCGAUGUGGUGUAAUGGUCAGCA	23
lmi-miR-candidate-49	CAUUACGAUUGAGAAGAAGGAA	22
lmi-miR-candidate-50	CGAGGCCCAUACUGCUGUGCAC	22
lmi-miR-candidate-51	CGACAGAUUAGUUGAGGAAACAAG	24
lmi-miR-candidate-52	ACUAUUCUGUUAGUGACACUGAA	23
lmi-miR-candidate-53	UUUCAGUCAUGCAAUCGUCAGC	22
lmi-miR-candidate-54	UUUCGCAGAUCUUCGCCACGUG	22
lmi-miR-candidate-55	UUGCCAGUAGGUGUGACAGAGAU	23
lmi-miR-candidate-56	AUGUGCCUGUCGCAUCUCGUCGU	23
lmi-miR-candidate-57	UGGGAACCUUACACAGGUGUCU	22
lmi-miR-candidate-58	UUUCAGUCACCCAAUCGUCAGCA	23
lmi-miR-candidate-59	GCCUCGGUAGCGCAGUAGUAGCG	23
lmi-miR-candidate-60	AGGUCCACCAAGCACUGGCUUA	22
lmi-miR-candidate-61	AGGGGUCGGGAGUUCGAUCCUCC	23
lmi-miR-candidate-62	UUAUUCUGUCCGUGCCUCGAAA	22
lmi-miR-candidate-63	UUGAGGCCAUGUAGUCAUCACU	22
lmi-miR-candidate-64	AUGAGCAAUGUUAUUCAAAUGG	22
lmi-miR-candidate-65	AGAGUGUCAAGAGCGCGACUC	21
lmi-miR-candidate-66	UGGACGGAGAUGAUAAGGUC	20
lmi-miR-candidate-67	AUUCUGUCGAUUUUCCGUGCAUA	23
lmi-miR-candidate-68	UGAAGCUCCUCAUAUCUGACCU	22
lmi-miR-candidate-69	UACAUCAUAGGUGUGCGGGUGU	22
lmi-miR-candidate-70	GGGUUGGUGGUGUAGUGGUA	20
lmi-miR-candidate-71	UGGAACACGAGGUAGAUUUGUC	22
lmi-miR-candidate-72	CAACAUGGCGGCGAACACGGGU	22
lmi-miR-candidate-73	GUUGAGCGAUGUAGAGACCCGG	22
lmi-miR-candidate-74	GUGAGAAGAUAGCACUCUAGUUG	23
lmi-miR-candidate-75	UUGAGAUCUCGAUUCCGUUACU	22
lmi-miR-candidate-76	CAAGGAAUCACUAAUCAUCCUA	22
lmi-miR-candidate-77	AGCCGUCGACCAUACUGAAUCG	22
lmi-miR-candidate-78	UGUCUACUUCCACUGAUCCCGCU	23
lmi-miR-candidate-79	CUGAAGUCACACGAGAGCGCCGU	23
lmi-miR-candidate-80	AGCAUGAUCAGUGGCAUGAAUU	22
lmi-miR-candidate-81	AGACUCAAUUUCUGGUCCUC	20
lmi-miR-candidate-82	UGUCUGUACAGUUUGCCGGAUC	22
lmi-miR-candidate-83	ACUGACUGCCCUAUUUCUUUGC	22

lmi-miR-candidate-84	GCCUCGGUAGCGCAGUAGGAGCG	23
lmi-miR-candidate-85	UGGACGGAGAUAUGAUAAGGA	21
lmi-miR-candidate-86	UGCGUUGUGAGUUGUCUUCGGCAU	24
lmi-miR-candidate-87	GAAGAGAUAGAGGAGUCAACUGC	23
lmi-miR-candidate-88	UUAAGGAGAAAUAUGCUGGAAAGG	24
lmi-miR-candidate-89	AGAGUGUCAGGAAUGUGAGUC	21
lmi-miR-candidate-90	UCUGUACCAGGAAUAUGAAUCG	22
lmi-miR-candidate-91	UGAUGCUGCAGGAGUUGUUGUGU	23
lmi-miR-candidate-92	AAUUGCUUGAUUCGUGCCGU	20
lmi-miR-candidate-93	UGUUAUUCUGAUUGACAGCUGU	22
lmi-miR-candidate-94	ACCAGAUUAAUGGAGGUAUGUGAA	24
lmi-miR-candidate-95	CUCUUUUGAAUAGUCUGACCCA	22
lmi-miR-candidate-96	CAAUGCCCUUGGAAAUCCCAA	21
lmi-miR-candidate-97	UUGAGAUCUCGCUUCCGUUACU	22
lmi-miR-candidate-98	UGCCUGGCGGCUUUAGCGCG	20
lmi-miR-candidate-99	UUGCGCAUCAGACGUCUGUGAGA	23
lmi-miR-candidate-100	AGGUUUUGAAGAGACAGCUGA	21
lmi-miR-candidate-101	UUGUGACCGCUGUGUUGUGCAU	22
lmi-miR-candidate-102	AGGCUCCAGUGAUGCGACGGUG	22
lmi-miR-candidate-103	UAGAAACGCACCCUUAGACCUA	22
lmi-miR-candidate-104	CAGUACUUGUGUCAUACUUUCA	22
lmi-miR-candidate-105	CAUCUUACCGGGCAGCAUUAU	21
lmi-miR-candidate-106	UUGAACACACACGGCUAUCACU	22
lmi-miR-candidate-107	UGGAAUAAAGAAGUAUGGAG	20
lmi-miR-candidate-108	ACUGACUUCUCCAUCUCUUUGC	22
lmi-miR-candidate-109	UCGAGCUGCUGGUGGGGUAACAU	23
lmi-miR-candidate-110	UCUGGUAUGUGGGUCACUUGCAA	23
lmi-miR-candidate-111	AUUAAGUGGAAAAGGAUGUG	20
lmi-miR-candidate-112	UUCUGGCUCAAUGCUCAGUCG	21
lmi-miR-candidate-113	UCAGGAAAUCAAUCGUGUAAGU	22
lmi-miR-candidate-114	UUGAACAUACACGGCCAUCACU	22
lmi-miR-candidate-115	UGACUGGGUGAUGCAACAGUUA	22
lmi-miR-candidate-116	UGUCCUAUCAGCGUCGGUCUUC	22
lmi-miR-candidate-117	GCCUGGUGAAGAUUUCGUCUGCAA	24
lmi-miR-candidate-118	UUACGUUCUCGGCUUUCCCU	20
lmi-miR-candidate-119	UUUCAGUCAUGCAAUCGUCACC	22
lmi-miR-candidate-120	UUCAUGGAAAGGUUCGAAGAGG	22
lmi-miR-candidate-121	UCUGGUAGGUGGGUCAGUCUCA	22
lmi-miR-candidate-122	AUACUGCUGUGCACUGCACCCAU	23
lmi-miR-candidate-123	AGAGUGCCAGGCGAGCAAUUCU	22
lmi-miR-candidate-124	CAACGGUUGCGGAGUUCCUUAA	22
lmi-miR-candidate-125	AUAUAGCUGUCCUGUCCGCGUU	22
lmi-miR-candidate-126	ACGAUUCUGAAGAUGUGACUGCAA	24

lmi-miR-candidate-127	GCCUGUCGCAUCUCGUCGCCACU	23
lmi-miR-candidate-128	UCACAGCCGCGCAAUCGUCAGC	22
lmi-miR-candidate-129	CUUCUGUGACUUAGUUGGAUGAU	23
lmi-miR-candidate-130	GGUCGGCGCAUAUGCAUCUGCA	22
lmi-miR-candidate-131	GUCUCUACCUGCUGCCUUGUCG	22
lmi-miR-candidate-132	GGGUUGAUGGUGUAGUGGUAU	21
lmi-miR-candidate-133	UCGCUAUUAUCUGUGACGAUAU	22
lmi-miR-candidate-134	UUUUAGUCACCCAAUCGUCAGA	22
lmi-miR-candidate-135	GCGAUGGUGUGUAAUGGUCAGCA	23
lmi-miR-candidate-136	GCGAUGGUCGUGUAAUGGUCAGCA	24
lmi-miR-candidate-137	CAUCCGGUCGGGAAACGGACGGU	23
lmi-miR-candidate-138	UCACAUACGCCCAAUCGUCAGCU	23
lmi-miR-candidate-139	UUCGGCGAUGAGAUCAGCCAGU	22
lmi-miR-candidate-140	UGAGUACCAGGUAUGUGAUUCA	22
lmi-miR-candidate-141	UUAGCAGAAGGUAAGUGACUCU	22
lmi-miR-candidate-142	UUUGAUUGCAUUUGAUCGUCGAA	23
lmi-miR-candidate-143	CUUCUGAGUGUUGGACAGAG	20
lmi-miR-candidate-144	UCCUGUUGCACCUGUUGGUUGAA	23
lmi-miR-candidate-145	GGGUUUGUUUUGUAUGGAGAGUGA	24
lmi-miR-candidate-146	UUCAUUGGCCUUGUGACACCU	21
lmi-miR-candidate-147	UCUCAGUUGUUGUAGGGACGGA	22
lmi-miR-candidate-148	UAGAGCUCCUUUAUUUGGGGAGGA	24
lmi-miR-candidate-149	ACGUGGCGACGAGAUGCGACAGU	23
lmi-miR-candidate-150	CAUCUGAGUGCUGGACAGAGGGU	23
lmi-miR-candidate-151	UUUCAUUCACGCAAUCGUCAGU	22
lmi-miR-candidate-152	UCUAUGCCACAUUGUCGUCACU	22
lmi-miR-candidate-153	ACACAUAUUCCUGAUACUGACA	22
lmi-miR-candidate-154	CUCUGUUCAGCACUCCGAGGGG	22
lmi-miR-candidate-155	UGUCUACCUCCACUGAUCCCGCA	23
lmi-miR-candidate-156	ACUAUUCUGUUAGUGACACUGU	22
lmi-miR-candidate-157	CGGCCUUCUCUGUAGGAGGUGG	22
lmi-miR-candidate-158	AGGAUCAGCGGAAGUGGACGGU	22
lmi-miR-candidate-159	GACGAUCGUUCUACAGUCCGAC	22
lmi-miR-candidate-160	UGUCUACCUCCACUGAUCCCGU	22
lmi-miR-candidate-161	CAACACAGGGAUUGUACAGGAU	22
lmi-miR-candidate-162	UCUCAGUUGUUGUAGGGAUGGA	22
lmi-miR-candidate-163	UGGAAUUAAAGAAGUAUGGAG	21
lmi-miR-candidate-164	AGACUCAACUUCUGGUCCUC	20
lmi-miR-candidate-165	UCAAAUCGUCGUAGCUGCUUU	21
lmi-miR-candidate-166	CCAGAAUCUUGGGGUCUGUGUCU	23
lmi-miR-candidate-167	CUCUGUUCAGCACUCUGAGGGG	22
lmi-miR-candidate-168	GUGAGAAAGCGGCAUUCUAGUUG	23
lmi-miR-candidate-169	UGUUGCUCUCAUCGUCAUCAUC	22

lmi-miR-candidate-170	UGGCAGGUACAUCUUUGAUGG	21
lmi-miR-candidate-171	AGGGAAUAGUGCUUUCUGAGUGGU	24
lmi-miR-candidate-172	GAUGGGUGUGGGUCUGGUGCAUG	23
lmi-miR-candidate-173	GCGAUGUUGUGUAAUGGUCAGCA	23
lmi-miR-candidate-174	GCGAUGGUUGUGUAAUGGUCAGCA	24
lmi-miR-candidate-175	UCCAUUGUAGUCUAGGUGGUU	21
lmi-miR-candidate-176	UCAUCCUGAGACUGUCUUUAU	21
lmi-miR-candidate-177	UCAUUUCAUCAGGCGGACCUUGG	23
lmi-miR-candidate-178	UGAGUACCAGGUAUGUGAUUCC	22
lmi-miR-candidate-179	GAAGAGAUAGGACAGUCAAUCU	22
lmi-miR-candidate-180	UCCUGGUCCACCACCUGGUUGA	22
lmi-miR-candidate-181	UUAGCAGAAGGUAAGUGACUCA	22
lmi-miR-candidate-182	UGUGUGUACUGUGGUGUUGAGGGU	24
lmi-miR-candidate-183	UGCCAGUCGGUGCAGUGCACAGU	23
lmi-miR-candidate-184	CAGAUUGCUUUCUGAUAUGAGG	22
lmi-miR-candidate-185	UUGCACUUCCACACACAUCACU	22

Table S4. The most abundant ten miRNA-like 5'-end small RNAs in the remaining reads after annotation of miRNAs, siRNAs and piRNA-like small RNAs.

Name	Dominant sequence	Length (nt)	Read	Percent of the dominant 5' end
miRNA-like-5'end-candidate-1	AAAACUACUUCUGCAUGACGCG	22	2104	83%
miRNA-like-5'end-candidate-2	UCGCACGUACUGUGCGAGCGGAU	23	1438	81%
miRNA-like-5'end-candidate-3	UCUAAAGGAUUUGUUACAGACUG	23	609	86%
miRNA-like-5'end-candidate-4	AAGCACAUUUCUGACACUGUCA	22	515	80%
miRNA-like-5'end-candidate-5	AUCGGAACAAGAUGGUCUAAUGC	23	384	85%
miRNA-like-5'end-candidate-6	UCGCACGCACUGUGCGAGCGGAU	23	379	85%
miRNA-like-5'end-candidate-7	UAGCUGCCUAGAGAAGGGCAUCA	23	292	84%
miRNA-like-5'end-candidate-8	UGGUAACUCCACCACCGUUGGCU	23	208	82%
miRNA-like-5'end-candidate-9	UGGUAACUCCACCACCGUUGGCG	23	179	85%
miRNA-like-5'end-candidate-10	AGACAUAUUCCUGAUACUGACA	22	138	88%

Table S5. Endo-siRNAs with different expression level between the two phases.

sequence	rpm (gregarious)	rpm (solitary)	fold(S/G)#
UAUCACUAUUGAACCACACGAUUCCGGG	1	54	54
GUGGGAGAAAUUCAGAAUUAAUGGCAA	1	25	25
UUCAUAAGUAGUUUGUAAGUUUAGGGAC	1	15	15
UUCAGACACGCACAGGGAGGAAAGAAG	1	13	13
UGAGAACGCAGAAAAUCCUCACGGACGGG	1	12	12
UCAGUAGUUUCAGCACGGACGCCAGAAG	1	10	10
UAACGAAAUCAUUCCACUGUAGGUGAAG	1	16	16
UCAAAUGUCUGUAAAUCUAUCGGACCAA	1	11	11
GUGGGAGAAAUUCAGAAUUAAUGGCAAA	1	10	10
AAAGUGAAGCAGAGCUCGCGCG	1	10	10
UAUAGGACCACAAUGCAGGACA	3	18	6
UACACGUGAACGAGAUGAGGAC	3	17	5.7
ACACGUGAACGAGAUGAGGACAU	2	12	6
UACUAAGGACGGCACAAAUAUCCAUGCC	5	28	5.6
UCCGCUCGACUUGGGCCCAGACCUCGGC	9	47	5.2
UCUGUUAACAUGACAAAUUCAAACGGCU	3	13	4.3
GUGGGAGAAAUUCAGAAUUAAUGGCA	8	30	3.75
AAAAGUGAAGCAGAGCUCGCGC	6	22	3.7
AAGUGAAGCAGAGCUCGCGCGG	3	12	4
AUACCUAUGUAGCACGCCGCGC	6	21	3.5
UAGUUUCAGCACGGACGCCAGAAGGGU	32	106	3.3
UAGUUUCAGCACGGACGCCAGAAGGG	8	25	3.1
UACAACUGUCAGACGCGCACGUGGUGC	41	76	1.85
UUUUUAAUCUGUGGAACGGUAUUGCAG	8	13	1.63
UAGUUUCAGCACGGACGCCAGAAGG	6	10	1.67
UGAGAACGCAGAAAAUCCUCACGGAC*	10	5	0.5
CAGGCACAUACAAAAGACACAC*	21	10	0.48
CAGGCACAUACAAAAGACACA*	13	4	0.31

*siRNAs with more abundance in the gregarious library

the rpm of the sequence in the solitary is divided by its rpm in the gregarious

Table S6. piRNA-like small RNAs with different expression level between the two phases.

sequence	rpm (gregarious)	rpm (solitary)	fold(S/G)#
CGGACAAGUACGGGGUGUCGGCGCUGA	2	125	62.5
UGAUGUAGCUCGAUGUCUCAGAUGCGUU	6	168	28
CGGACAAGUACGGGGUGUCGGCGCUG	3	53	13.3
UACAAAAGCUAGAAAUGUAGGUUUGCCU	2	24	12
UGAUGUAGCUCGAUGUCUCAGAUGCGU	3	17	5.7
CGGACAAGUACGGGGUGUCGGCGCU	26	83	3.2
CGGACAAGUACGGGGUGUCGGCGC	15	31	2.1
UGCAUAGGGCAGGUUUUACAACGGG*	21	7	0.3

*piRNAs with more abundance in the gregarious library

the rpm of the sequence in the solitary is divided by its rpm in the gregarious

Methods

Evaluation of the method for identifying locust-specific miRNAs

In order to identify locust-specific miRNAs in the absence of its genomic data, a new method was developed based on biogenesis of miRNA. After summarizing the features of the released miRNAs, some principles were used to search the non-conserved miRNA candidates in the small RNA library of the locust. By analyzing public database of fly miRNAs sequenced by 454 [20], we found the star sequences of 131/149 (88%) miRNAs detected in the high-throughput small RNA library. So it is possible to find novel miRNAs in the library by searching miRNA:miRNA* duplexes. The rules adopted to find candidate miRNA-duplex-like pairs were listed in the methods. When being searched in the pool composed of the 131 fly miRNA and their corresponding miRNA* sequences, 70 miRNA duplexes could be found, which indicated the feasibility of our method for finding miRNA duplex. Moreover, seven locust-conserved miRNAs whose star sequences were validated could also be found, indicating that more than half of the locust-specific miRNAs could be discovered if our criterion for searching novel miRNAs was applied.

To examine whether those miRNA duplex-like pairs were canonical miRNA duplexes or not, we joined the two sequences in each candidate pairs with a standard hairpin-forming linker sequence (GCGGGGACGC) followed by secondary structure prediction by mfold [22, 23]. Canonical miRNA precursor can fold to typical hairpin structure with lower free energy. We analyzed 70 miRNA duplexes of the fly detected in our method and found that 96% of the pairs linked by the linker sequence had $\triangle G$

lower than -21kcal/mol. So the candidate pairs of the locust with △G higher than or equal to -21kcal/mol were discarded. Moreover, when searching the database in our method, we found there might be more than one base-pair partners for one small RNA sequence. In order to choose the genuine duplexes, we studied the features of canonical miRNA duplexes of the fly when we merged them into our locust library. Although 43 fly miRNAs had other partners besides their own stars when we searched in the library, the genuine star sequences of 88% of the 43 were those owning the lowest free energy when being folded by mfold. So if one sequence in the locust library had more than one base-pair partners, that with the lowest free energy was considered as the genuine one. The pairs with bulge bigger than 6nt and multiple loops were also discarded. Based on the rules we proposed, some locust-specific miRNAs could be found although there was no locust genomic sequence data available.
